# Secondary structure RNA elements control the cleavage activity of DICER

**DOI:** 10.1038/s41467-022-29822-3

**Published:** 2022-04-19

**Authors:** Trung Duc Nguyen, Tam Anh Trinh, Sheng Bao, Tuan Anh Nguyen

**Affiliations:** Division of Life Science, The Hong Kong University of Science and Technology, Hong Kong, China

**Keywords:** Enzyme mechanisms, Small RNAs

## Abstract

The accurate and efficient cleavage of shRNAs and pre-miRNAs by DICER is crucial for their gene-silencing activity. Here, we conduct high-throughput DICER cleavage assays for more than ~20,000 different shRNAs and show the comprehensive cleavage activities of DICER on these sequences. We discover a single-nucleotide bulge (22-bulge), which facilitates the cleavage activity of DICER on shRNAs and human pre-miRNAs. As a result, this 22-bulge enhances the gene-silencing activity of shRNAs and the accuracy of miRNA biogenesis. In addition, various single-nucleotide polymorphism-edited 22-bulges are found to govern the cleavage sites of DICER on pre-miRNAs and thereby control their functions. Finally, we identify the single cleavage of DICER and reveal its molecular mechanism. Our findings improve the understanding of the DICER cleavage mechanism, provide a foundation for the design of accurate and efficient shRNAs for gene-silencing, and indicate the function of bulges in regulating miRNA biogenesis.

## Abstract (web summary)

MicroRNA precursors are cleaved by DICER to generate mature microRNAs in the cytoplasm. Here the authors employ high-throughput analysis of DICER cleavage activity and identify RNA secondary elements in precursor miRNAs and shRNAs, including a single nucleotide bulge, which govern its cleavage efficiency and accuracy.

## Introduction

MicroRNAs (miRNAs) are small RNAs of 21–22 nt, which play a vital role in gene regulation. miRNAs make base pairs with their target mRNAs, and in this way either trigger mRNA degradation or translational suppression^1–3^. At the start of miRNA biogenesis, primary miRNAs (pri-miRNAs) are synthesized by RNA polymerase II in the nucleus. These are then processed by Microprocessor to generate precursor miRNAs (pre-miRNAs), which are exported to the cytoplasm by Exportin-5. In the cytoplasm, the pre-miRNAs are cleaved by DICER to generate miRNA duplexes of 21–22 nt^4–6^. A protein called Argonaute (Ago) takes one strand of each miRNA duplex (to generate an Ago-miRNA) for maturation, whereas the other strand is discarded^3,7^. The Ago-miRNA complex makes up the core of the miRNA-induced silencing complex (RISC), which is responsible for the mRNA degradation or translational suppression of its target mRNAs^3,4,8^. The expression and thus functions of miRNAs are therefore largely dependent on the activities of these miRNA biogenesis factors.

**Fig. 1:**
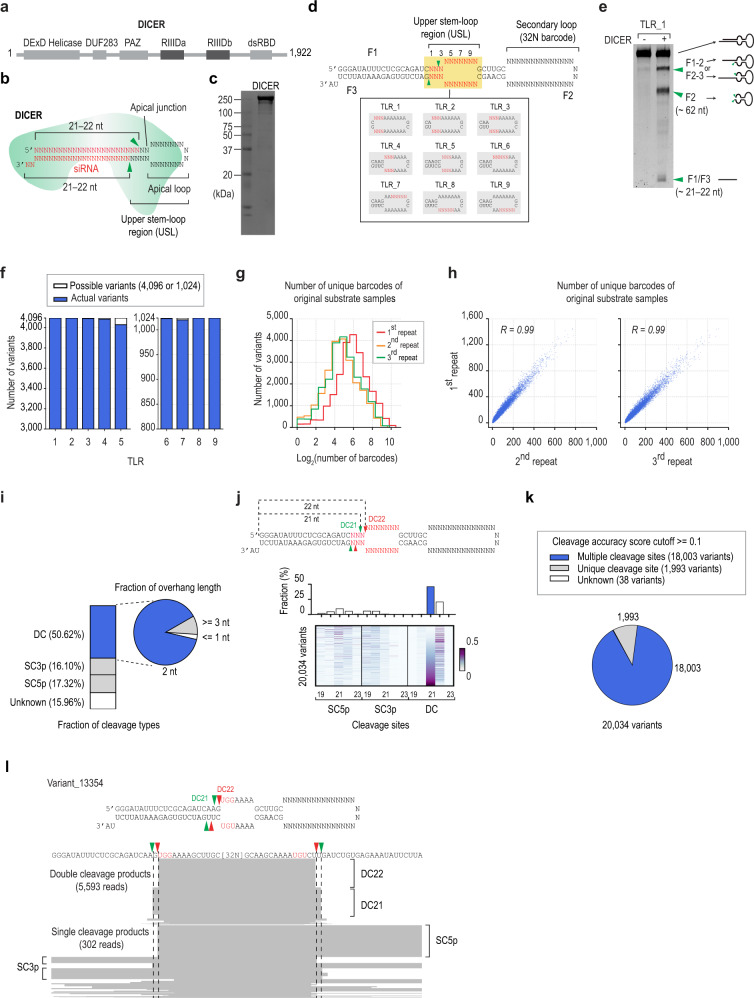
High-throughput DICER cleavage assays. **a** The protein domain structure of DICER. DExD helicase domain, DUF283: domain of an unknown function, PAZ: Piwi/Argonaut/Zwille, RIIIDa and RIIIDb: RNase III domains, and dsRBD: double-stranded RNA-binding domain. **b** Schematic of shRNA. The siRNA region is red, and the green arrowheads indicate the DICER cleavage sites. **c** The purified DICER. **d** The randomized artificial shRNAs. This two-loop shRNA (TLR) contained primary and secondary loops. The upper stem-loop region (USL) was randomized in the 3-base pair or 5-nt windows. The secondary loop contained 32-nt randomized sequences (32N), which served as barcodes. **e** The high-throughput (HT) shRNA cleavage assay. DICER cleaved the artificial shRNA (subgroup TLR_1) into F1-2, F2-3, F1, F2, and F3 fragments. **f** Identification of the synthesized shRNA variants in the HT cleavage assays by next-generation sequencing (NGS). **g** The unique barcodes of each shRNA variant were counted from three repeated HT assays. The distribution of log_2_(number of unique barcodes) are shown. **h** The reproducibility of the HT cleavage assays. R is Pearson’s correlation coefficient. **i** The fractions of different cleavage types (single or double cleavages) and different overhang lengths of the double cleavage products in the DICER-cleaved products. **j** The different cleavage sites of DICER on the shRNA variants. Upper panel: The fraction of variants processed at the major cleavage sites. The ‘major’ cleavage site of a variant is the cleavage site with the highest cleavage accuracy score. Lower panel: The cleavage accuracy score of 20,034 recovered variants in the HT cleavage assays at different cleavage sites. The cleavage accuracy score is colored according to the color scale. **k** The inaccuracy of DICER in processing shRNAs. The fraction of variants with unique or multiple cleavage sites. The SC3p, SC5p, and DC cleavage sites are from 19 to 23, as shown in (**j**). Cleavage sites with a cleavage accuracy score ≧0.1 were selected. **l** The double and single cleavage products of a representative variant are shown. Randomized nucleotides are in red. DC: double cleavage, SC5p: single cleavage on the 5p-strand, SC3p: single cleavage on the 3p-strand. Source data are provided as a Source Data file.

DICER is an RNase III enzyme. It consists of two RNase III domains (RIIIDa and RIIIDb) that form an intramolecular heterodimer, which cleaves the two strands of pre-miRNAs^9–14^ (Fig. 1a). DICER also has a double-stranded RNA binding domain (dsRBD), located at the C-terminal region. The dsRBD contains RNA-binding affinity and enhances the cleavage efficiency of DICER, and does not interfere with its cleavage sites^13,15,16^. In addition, the dsRBD is vital for the nuclear localization of DICER^17^. DICER also contains a PAZ domain (located in the middle region), which includes binding pockets for the 5p- and 3p-ends of pre-miRNAs, and this plays a critical role in determining the cleavage sites of DICER. Using these two pockets, DICER locates its catalytic center ~21–22 nt away from the ends of the pre-miRNAs^11,12,15,18,19^ (Fig. 1b). The apical loop (or the internal loop close to the apical loop) also plays an important role in governing the cleavage sites of DICER. It is reported that DICER could find the cleavage sites ~2 nt from the apical or internal loop on several tested substrates^20^. The N-terminal region of DICER has three tandem RNA helicase domains. The RNA helicase domain is thought to interact with the loop of the pre-miRNA^10,20,21^. The upper stem-loop region (USL) of pre-miRNAs (Fig. 1b) also seems essential for DICER cleavage^21–23^; however, it is still largely unknown what specific RNA elements of the USL are favorable or unfavorable for this process. For this reason, it is of critical importance to investigate how different sequences and structures of the USLs control the DICER cleavage mechanism.

The ability of DICER to cleave pre-miRNAs is used in short-hairpin RNA (shRNA)-based gene silencing technology. Here, DICER is utilized to cleave shRNAs (i.e., pre-miRNAs mimics), to generate shRNA-resulting siRNAs for mediating gene silencing^24–29^. shRNAs have been widely used in research, and a number have also been developed into biomedical therapies. These include CCR5-targeting shRNAs for the treatment of human immunodeficiency virus (HIV) disease, PD-L1-targeting shRNA CAR-T for the lung cancer treatment, and FURIN-targeting shRNAs for reducing the number of autologous tumor cells^28,30,31^. shRNAs can either be synthesized in vitro and then delivered to cells or generated in vivo via the action of RNA polymerases, which transcribes shRNA-coding DNAs via an appropriate plasmid transfected into cells^32–34^. A typical shRNA consists of a lower stem of ~21–22 bp, an siRNA sequence targeting a specific mRNA using the base-pairing mechanism, and a USL containing a few base pairs and a loop of ~6 nt^20,24,35^. Since we do not fully know which RNA elements in the USL of pre-miRNAs and shRNAs facilitate their cleavage by DICER, in current shRNA designs used for shRNA-based gene silencing, the USL has not been optimized.

The cleavage activity of DICER is critical for the knockdown (KD) efficiency of shRNAs in human cells. In this study, we aimed to identify any RNA elements in the USL region of shRNAs that facilitate this cleavage and thus enhance the KD efficiency of shRNAs. One of the main problems in studying the USL region is that DICER cleavage sites might occur within the USL. This makes it impossible to determine which cleaved products are derived from a particular sequence of the original shRNA substrate. Here, we designed shRNAs containing two loops (TLR). The primary loop served as the USL region for DICER to interact, whereas the secondary loop possessed 32-nt randomized barcode sequences (32N), which allowed the cleaved-DICER products and their original substrates to be matched after the cleavage reaction was complete. Using this 2-loop shRNA-designed system, we set up high-throughput (HT) enzymology assays using the purified DICER enzyme and tens of thousands of shRNA sequences, which contained the randomized USL regions. Analysis of the HT cleavage assays allowed us to identify the optimal USL structures and sequences for DICER cleavage. For example, bulges in the 22 position (22-bulges) on the 3p-strand of shRNAs enhanced DICER activity and thus increased the KD efficiency of shRNAs. In addition, we also demonstrated that the 22-bulges, which are present in many pre-miRNAs, are critical for DICER to produce miRNAs accurately and efficiently from pre-miRNAs during miRNA biogenesis. Furthermore, we demonstrated the single cleavage (SC) activity of DICER on either the 5p- or 3p-strand and revealed its molecular mechanism. This study extends our understanding of the DICER cleavage mechanism and explains the roles of identified RNA elements in regulating miRNA biogenesis. Furthermore, it provides an alternate approach for designing effective shRNAs that exhibit a high KD efficiency.

## Results

### High-throughput DICER cleavage assays

We purified DICER from human cells (Fig. 1c) and tested its cleavage activity with several pre-miRNAs, including pre-mir-30a and pre-mir-92a-2. The purified DICER cleaved these pre-miRNAs and generated miRNAs of ~21–22 nt, demonstrating its usual cleavage activity (Supplementary Fig. 1a–c).

DICER typically cleaves shRNAs and pre-miRNAs substrates, generating F1, F2, and F3 fragments. The ends of the F2 fragment allowed us to identify the cleavage sites of DICER (Supplementary Fig. 1d). However, if the regions between the F1 and F2 or F2 and F3 junctions are variable, then it is not feasible to determine which F2 sequences are derived from which F1-F2-F3 shRNA combinations. For example, if the nt sequence around the cleavage sites were randomized, the final F2 sequence could result from many combinations of different F1 and F3 sequences. Therefore, it is not possible to conduct HT cleavage assays for DICER with shRNAs containing randomized USL sequences. To solve this problem, we synthesized an artificial two-loop shRNA structure containing the stem region as well as the primary and secondary loops. The stem sequence, which does not map to any region in the human genome, was referred to the artificial pri-miRNA used in the previous report^36^. Four G-C pairs were intentionally added in the constant region between two loops to stabilize its base pairing. The second loop containing the barcodes was distant from cleavage sites, and thus it should not interact with DICER^9,10,20^. The primary loop served as the USL region for DICER to interact, whereas the secondary loop functioned as a 32-nt randomized barcode sequence. This TLR structure helped us determine which of the cleaved products resulted from which of the original shRNA sequences.

We randomized the USL region of the TLR by introducing three randomized base pairs in positions 1 to 5 (subgroups, TLR_1 to TLR_5), or a 5-nt randomized single-stranded RNA (ssRNA) region on either the 5p- or 3p-strand (TLR_6 to TLR_9) (Fig. 1d). shRNA variants produced in subgroups 1–5 expectedly contained different base pairs, mismatches, and bulges in the upper stem region, while those generated from subgroups 6–9 possessed different primary sequences in the loop. We found that DICER cleaved TLR_1 and generated an siRNA (F1/F3) of ~21–22 nt and an ssRNA (F2) of ~62 nt containing the two loops (Fig. 1e). This indicates that DICER exhibited its typical RNase III activity on the artificial shRNAs. The longer cleaved fragments displayed as F1-2 and F2-3 were later identified as the SC products of DICER. We then conducted HT cleavage assays for DICER and each of the nine randomized shRNA subgroups above. Subsequently, we cloned the cleaved products (F2, F1-2, and F2-3) and the original shRNAs, and generated DNA libraries, which were subsequently sequenced by next-generation sequencing (NGS) (Supplementary Fig. 1e–g).

We collectively generated 23,207/23,296 shRNA variants from the three repeated HT cleavage assays and obtained 98.34%–100% of the expected variants for each subgroup (Fig. 1f). In total, 99.62% of all the expected shRNA variants were obtained. We then determined the raw count or barcode numbers for each variant in the original substrate samples of three repeated assays. The median unique barcode numbers were 59, 25, and 29, and the median values of the raw counts were 121, 38, and 45 for the three repeats (Fig. 1g and Supplementary Fig. 1h). We also found that the unique barcode numbers and raw counts for each variant were highly reproducible among the three repeats (Fig. 1h and Supplementary Fig. 1i).

### Multiple cleavage modes of DICER on shRNAs

We analyzed the sequences of the cleaved fragments (F2 and the longer fragments, F1-2, F2-3; Fig. 1e and Supplementary Fig. 1e) from the NGS data and determined the cleavage sites of DICER on the shRNAs. First, we found that DICER exhibited the typical cleavage activity of RNase III enzymes by releasing most of the cleaved products containing a 2-nt overhang at their 3p-ends. However, DICER produced 1-nt or 3-nt overhang products from a small fraction of the shRNAs (Fig. 1i). In addition, we found that DICER cleaved most shRNAs by two cleaving methods, SC and double cleavage (DC). The SC was consistent with what was observed with several human pre-miRNAs^37^ and reflected a feature of RIIIDa and RIIIDb that could cleave dsRNA independently of each other^13^. The DC occurred at multiple sites, ranging between 19 to 23 base pairs from the first base pair of the stem (Fig. 1j, k). However, the enzyme preferred cleaving shRNAs mainly at DC21 and DC22, 21 and 22 base pairs, respectively, from the first base pair of the stem (Fig. 1j–l). DICER SC occurred on either the 5p- or 3p-strand in similar positions as the DC (Fig. 1j, l).

### The distinct cleavage activities of DICER on different structural shRNAs

**Fig. 2:**
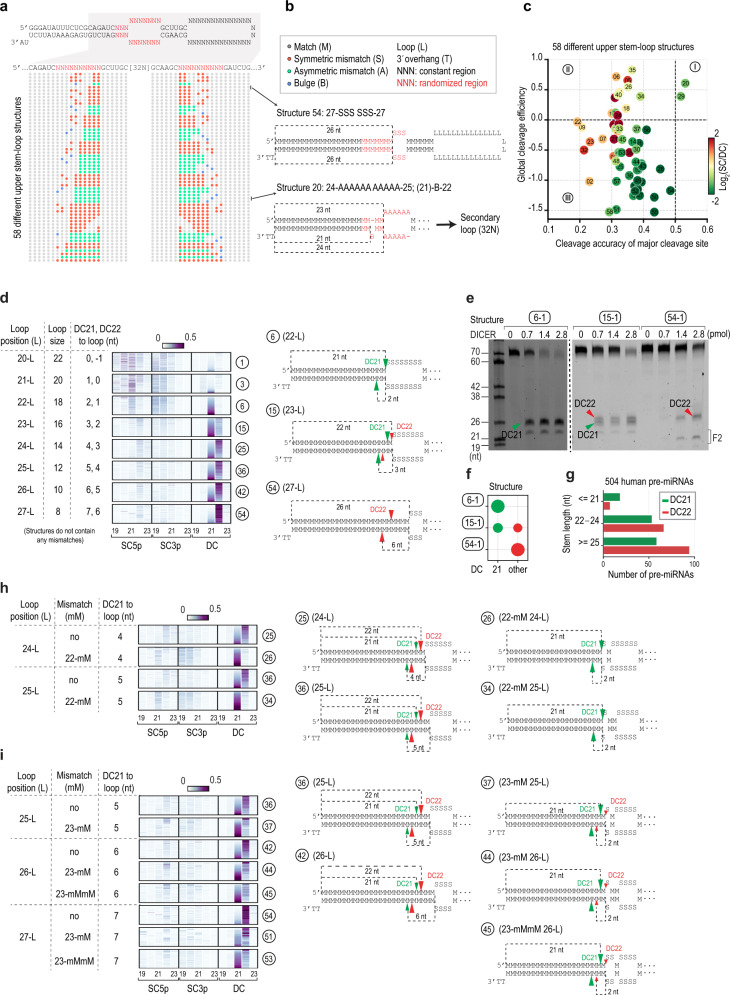
The cleavage activity of DICER on different upper stem-loop structures. **a** The positional structural profile of 58 shRNA structures, each had at least 10 variants identified in the HT cleavage assays. We predicted the secondary structure of 20,034 shRNA variants using RNAfold^62^. The dot-bracket structure, obtained from RNAfold, was converted into custom-designed structures in which each nt was assigned one of the following letters: L (loop), M (match), S (symmetric mismatch), A (asymmetric mismatch), B (bulge), and T (3’-overhang). The grey, red, green, and blue circles represent M, S, A, and B’s positional structure. The randomized regions are shown in red. **b** Two representative shRNA structures. **c** Scatter plot of 58 shRNA structures comparing the global cleavage efficiency and the cleavage accuracy of the major cleavage sites, as well as the SC/DC ratios. The log_2_(SC/DC) values were colored according to the color bar. The number inside each circle indicates the name of each structure as shown in (**a**, **b**) (Supplementary Data 1). Fifty-eight shRNA structures were further classified into I, II, and III groups. **d** The cleavage accuracy scores of DICER on shRNAs containing various distances between the major cleavage site and the loop. The cleavage accuracy score at the different cleavage sites on each variant was measured as described in the Methods. **e** The in vitro DICER cleavage assays of the variants containing structures 6, 15, or 54 (Supplementary Data 2, Supplementary Fig. 2b). The assays were repeated three times. **f** The DICER cleavage accuracy in (**e**) was calculated as the ratio of the cleaved products at a certain position to those at other positions. **g** The cleavage sites of DICER on human pre-miRNAs containing different stem lengths. **h** The cleavage accuracy score of DICER at DC21 on shRNAs having no mismatch or a single mismatch at 2 nt from DC21. **i** The cleavage accuracy score of DICER on shRNAs containing no mismatch, a single mismatch, or double mismatches at 2 nt from DC22. Source data are provided as a Source Data file.

Since DICER showed inconsistent cleavage activities on shRNA variants containing randomized USLs, the sequences and structures of the USLs might significantly impact on the cleavage activity of the enzyme. We defined the secondary structures of the shRNAs using six features: L (loop), M (match), S (symmetric mismatch), A (asymmetric mismatch), B (bulge), and T (3'-overhang). Based on these various feature elements, all the variants identified in the HT assays were clustered into 97 groups such that each group (or structure) had a distinct secondary USL structure. Of these 97 structures, we selected 58, which contained more than 10 variants (identified from the HT assays) for further analysis (Fig. 2a, b; Supplementary Data 1). In brief, we measured the global cleavage efficiency, accuracy, and SC/DC ratios as the ratios of the products cleaved at all cleavage site(s) to the original substrate, the products cleaved at each cleavage site to the total products cleaved at all positions, the total SC products to total DC products, respectively. We demonstrated that each structure showed distinct cleavage efficiency, cleavage accuracy, and SC/DC ratio (Fig. 2c) and the SC/DC ratio seemed to be moderately correlated with the cleavage efficiency (Supplementary Fig. 2a). We subsequently validated the accuracy and efficiency of the DC for 9 structures in groups I, II, and III (Supplementary Fig. 2b–e). Unlike the global cleavage efficiency shown in Fig. 2c, we did not include the SC products in measuring the DC efficiency since they were not apparent in the gel. Consistent with the HT cleavage assays, we found that DICER cleaved structure 20 in group I with the highest accuracy and efficiency than any structures in groups II and III (Supplementary Fig. 2b–e).

### The mismatch and loop positions affect DICER cleavage sites

The loops located 2 nt from DC21 significantly increased DC21 cleavage (Fig. 2d, structure 6). This is consistent with a previous study, which showed that for several transfected shRNAs, the apical loop or an internal loop 2 nt from the cleavage sites enhanced the cleavage efficiency in this location^20^. However, we observed that loops that were 2 nt from DC22 did not significantly increase DC22 cleavage; most of the 2 nt-distant loop-containing shRNAs were still cleaved at DC21 (Fig. 2d, structure 15). This indicate that the loop counting rules might only work efficiently for DC21 and not for DC22. Next, we demonstrated that more distant loops from the DICER cleavage sites reduced DC21 but facilitated DC22 (Fig. 2d, structures 25, 36, 42, and 54). We then validated this observation from the HT cleavage assays with the structures 6, 15, and 54 that contained the different stem sequences (Fig. 2e, f, Supplementary Fig. 2f–h). We then analyzed cleavage by DICER on human pre-miRNAs and found that the enzyme preferred cleaving long-stem pre-miRNAs at DC22 (Fig. 2g). In this analysis, the cleavage sites of DICER on human pre-miRNAs were determined by the 3p-end of 5p-miRNAs, as described in detail in the Methods section. In addition, we observed that DICER cleaved the 2-nt distant loop-containing shRNAs similarly regardless of loop size (Supplementary Fig. 2i), and the 2-nt distant loops strongly influenced the DC21 cleavage sites on shRNAs sharing the same-sized loops (Supplementary Fig. 2j). These results showed that it is the position rather than the size of the loop that affects the cleavage accuracy of DICER at DC21.

Interestingly, we found that a single mismatch located 2 nt from the DC21 cleavage also significantly enhanced DICER cleavage at DC21 (Fig. 2h, Supplementary Fig. 2k–m). In contrast, single and double mismatches located 2 nt from DC22 did not increase DC22 cleavage (Fig. 2i). Together, these findings indicate that the position of the loop or single mismatch has different effects on DC21 and DC22.

### The 22-bulge enhances DICER cleavage activity in HT assays

**Fig. 3:**
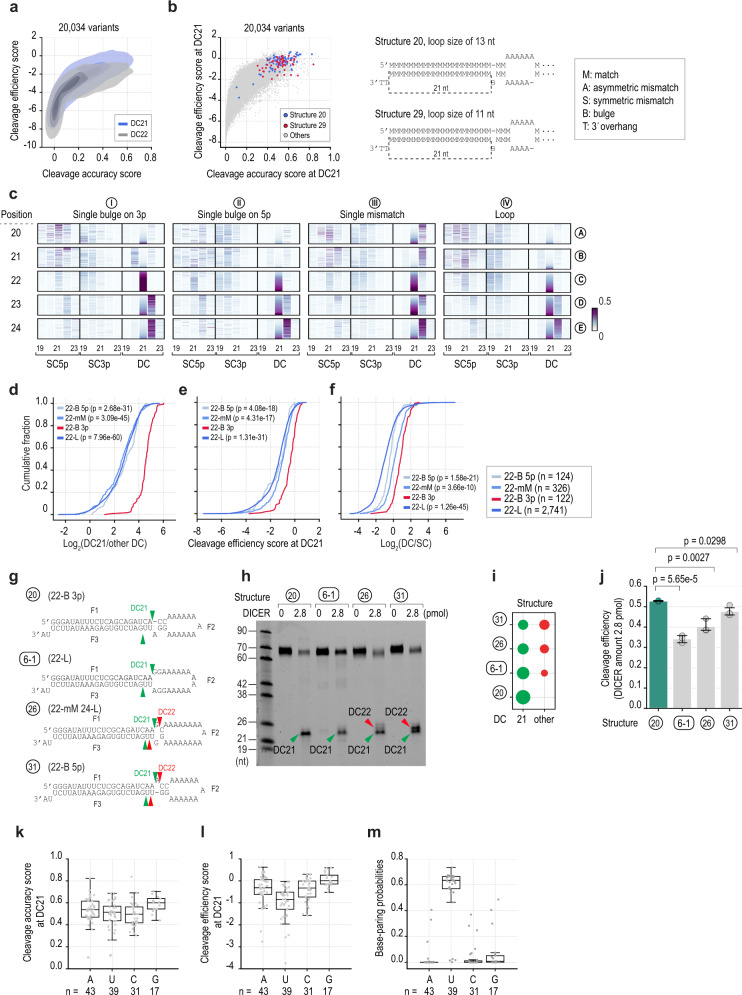
The 22-bulge governs the accuracy and efficiency of DICER cleavage at DC21. **a** The cleavage efficiency and accuracy scores of DICER in the HT cleavage assays. Density plots of DC21 and DC22 are shown in blue and grey, respectively. **b** The 22-bulge-containing structures (structures 20 and 29) exhibited the highest cleavage accuracy and efficiency at DC21. **c** The cleavage accuracy scores of DICER in shRNAs containing different structures. **d**–**f** Line graphs to show the log_2_(DC21/other DC) (**d**), cleavage efficiency score at DC21 (**e**), and log_2_(DC/SC) (**f**) of variants containing: 22-bulge 3p (22-B 3p), 22-loop (22-L), 22-single mismatch (22-mM), or 22-bulge 5p (22-B 5p). The p-values were calculated by two-sided Wilcoxon rank-sum tests. **g** The structures and sequences of shRNAs. **h** The in vitro DICER cleavage assays for RNAs in (**g**). **i** The cleavage accuracy of DICER in (**h**) was calculated as a ratio of the cleaved product at a certain position to those at other positions. **j** The cleavage efficiency of DICER in (**h**) was calculated as a ratio of the cleaved product at DC21 or DC22 to the original substrate, *n* = 3 independent experiments. A two-tailed t-test calculated the p-values. The error bars were presented with 95% confidence intervals. **k**, **l** The cleavage accuracy and efficiency scores of the 22-bulge variants containing different nt in the bulge. **m** The base-pairing probabilities of different nt in 22-bulge generated by RNAfold^62^. The positional base-pairing probability of a variant was calculated as the ratio of the number of structures containing a base-pair at a specific position to the total number of predicted structures. In (**k**–**m**), The number of variants for each nt: *n* = 43 (A); *n* = 39 (U); *n* = 31 (C); *n* = 17 (G). The center line is median, the lower and upper bounds of the box are the 25^th^ and 75^th^ percentiles, whiskers show 1.5x the interquartile extending from bounds of box, minima is the minimum value, and maxima is the maximum value. Individual data values are shown as dots. Source data are provided as a Source Data file.

The above findings showed that DICER mainly cleaves shRNAs at DC21 and DC22. We further showed that DICER cleaves shRNAs at DC21 more efficiently and accurately than DC22 (Fig. 3a). We then focused on the variants that displayed the highest cleavage activity at DC21 and found that most of these contained a single-nucleotide bulge in the 22-position on the 3p-strand (hereafter called the 22-bulge) (Fig. 3b). The effect of the 22-bulge was not significantly affected by the size or position of the apical loop (Supplementary Fig. 3a, b). Next, we demonstrated that single-nucleotide bulges in locations other than the 22-position on the 3p-strand (Fig. 3c, panel I) did not increase the accuracy of DICER at DC21. Interestingly, we found that 22-bulge-containing shRNAs were cleaved with much higher accuracy (Fig. 3c, compare panel I-C with panel II-C, III-C, or IV-C; Fig. 3d) and efficiency (Fig. 3e) at DC21, when compared with a 22-bulge on the 5p-strand, 22-mismatch, or 22-loop-containing shRNAs. In addition, the 22-bulge also enhanced the DC/SC ratio more than the other elements (Fig. 3f). The above observations were also validated in the cleavage assays (Fig. 3g–j). We then compared the cleavage activity of DICER on the 22-bulge variants, which had different nucleotide identities in the 22-bulge. Our data demonstrated that uridine (U) in the 22-bulge was the least effective than other nucleotides, G, A, and C, in stimulating the DICER cleavage (Fig. 3k, l). This lower activity of U could be partially explained by the fact that U in the bulge was found to have a higher alternative base-pairing probability with the 5p-strand than the other nucleotides (Fig. 3m).

### Verification of the 22-bulge effect on numerous shRNA sequences

**Fig. 4:**
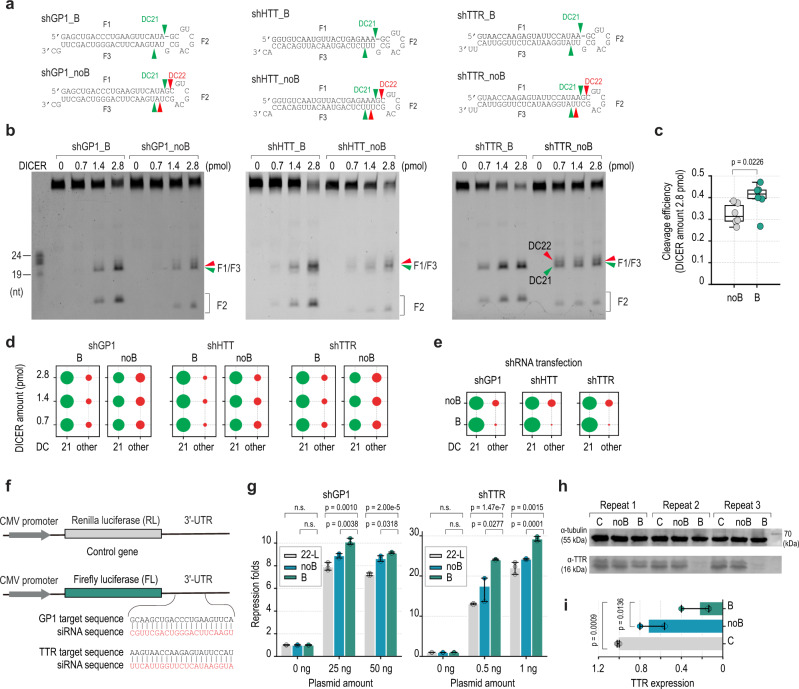
The 22-bulge enhances DICER activity and the knockdown efficiency of shRNAs. **a** The 22-bulge (B) and nobulge-shRNAs (noB) contain different siRNA sequences and share the same USLs. **b** In vitro DICER cleavage assays for the 22-bulge and nobulge shRNAs. **c** The cleavage efficiency of DICER on the 22-bulge and nobulge shRNAs was shown in (**b**) and Supplementary Fig. 4b,*n* = 6 independent experiments. The p-values were calculated by two-sided Wilcoxon rank-sum tests. The center line is median, the lower and upper bounds of the box are the 25^th^ and 75^th^ percentiles, whiskers show 1.5x the interquartile extending from bounds of box, minima is the minimum value, and maxima is the maximum value. Individual data values are shown as dots. **d** The cleavage accuracy of DICER in (**b**) was calculated as a ratio of the cleaved products at a certain position to those at other positions. **e** Confirmation of DICER cleavages in human cells. The siRNAs resulting from cellular shRNAs were sequenced by NGS. **f** Construction of the firefly luciferase (FL) reporter gene and shRNA design. **g** The 22-bulge shRNAs (B) had higher gene-silencing activity than the nobulge shRNAs (noB) or 22-loop shRNAs (22-L) in reporter assays. These dual-luciferase reporter assays were repeated three times. A two-tailed t-test calculated the p-values. The error bars were presented with 95% confidence intervals. **h** The 22-bulge shRNAs silenced the expression of the TTR of endogenous genes more efficiently than did nobulge shRNAs. The western blot experiments were repeated three times. **i** Bar graphs showing the knockdown efficiency of the 22-bulge-shRNA and nobulge-shRNA on the expression of TTR protein, *n* = 3 independent experiments. A two-tailed t-test calculated the p-values. The error bars were presented with 95% confidence intervals. Source data are provided as a Source Data file.

Next, we designed six different shRNAs. Each contained a unique stem (siRNA) sequence, but they all shared the same 22-bulge-containing USL structure (Fig. 4a and Supplementary Fig. 4a). We compared the cleavage activity of DICER for each pair of 22-bulge- (B) and nobulge- (noB) shRNAs and found that the 22-bulge increased the cleavage efficiency and accuracy of the shRNAs when tested in the in vitro cleavage assays (Fig. 4a–d, and Supplementary Fig. 4b–d). In addition, we showed that the 22-bulge also increased cleavage of the DICER-TRBP complex at DC21 (Supplementary Fig. 4e). We then transfected plasmids expressing shRNAs into human HCT116 cells and conducted small RNA sequencing. We confirmed that the 22-bulge-containing shRNAs produced more accurate DC21 cleaved products than the nobulge-containing shRNAs (Fig. 4e and Supplementary Fig. 4f). These data showed that the 22-bulge could enhance the cleavage efficiency and accuracy of DICER cleavage regardless of the stem sequence.

### The 22-bulge increases the knockdown efficiency of shRNAs

We examined the knockdown (KD) efficiency of the 22-bulge-shRNAs (B), nobulge-shRNAs (noB), and 21bp-stem-shRNAs (22-L) (which shared the same siRNA sequence), in dual-luciferase reporter assays. The targeting region of each shRNA was introduced in the 3'-UTR of the firefly luciferase reporter gene (Fig. 4f), the reporter and shRNA-expressing plasmids (or synthetic shRNAs) were transfected into the HEK293T cells. We demonstrated that the shRNA_B had a higher KD efficiency than the shRNA_noB and shRNA_22-L (Fig. 4g and Supplementary Fig. 4g). This was consistent with the in vitro cleavage assays showing that DICER cleaved the shRNA_B better than shRNA_noB and shRNA_22-L (Fig. 4b–d and Supplementary Fig. 4b–d, h–j)

Next, we transfected the 22-bulge or nobulge shRNAs into the HepG2 cells and tested their ability to knock down TTR expression. We demonstrated that the 22-bulge shRNAs knocked down the expression of TTR more efficiently than the nobulge-shRNAs (Fig. 4h, i).

### The 22-bulge controls the cleavage activity of DICER in pre-miRNAs

**Fig. 5:**
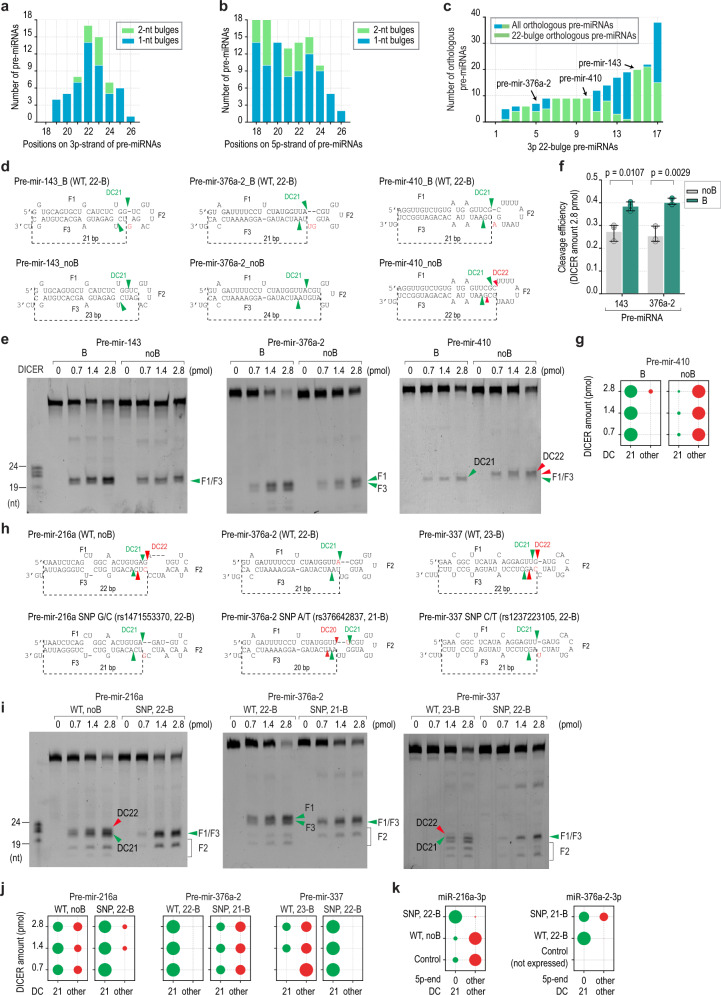
The 22-bulge controls the cleavage activity of DICER on human pre-miRNAs. **a**, **b** Human pre-miRNAs contain a bulge in different positions on their 3p-strand (**a**) or 5p-strand (**b**). The pre-miRNA sequences were folded using RNAfold, and the number of pre-miRNAs containing a bulge in positions 18–26 from their 5p-end was quantified. **c** The number 59 of pre-miRNA orthologs containing a 22-bulge was determined. **d** The structures and sequences of 22-bulge pre-miRNAs and their nobulge variants. **e** The in vitro DICER cleavage assays of the 22-bulge and nobulge variants of human pre-miRNAs. **f** The cleavage efficiency of DICER on the 22-bulge and nobulge pre-miRNAs shown in panel (**d**), *n* = 3 independent experiments. The cleavage efficiency of DICER was calculated as the ratio of the cleaved product at DC21 or DC22 to the original substrate. A two-tailed t-test calculated the p-values. The error bars were presented with 95% confidence intervals. **g** The accuracy of DICER cleavage was calculated as the ratio of the cleaved product at DC21 to that at different positions. **h** The structures and sequences of the 22-bulge pre-miRNAs and their SNP variants. **i** The in vitro DICER cleavage assays of the 22-bulge and their SNP pre-miRNA variants. **j** The accuracy of DICER cleavage was calculated as the ratio of the cleaved product at DC21 to that at different positions. **k** Confirmation of the DICER cleavages on the pre-miRNAs by 5p-end of 3p-miRNAs. The sequences of miRNAs resulting from the ectopically expressing pre-mir-216a and pre-mir-376a-2 were determined by NGS. Source data are provided as a Source Data file.

We analyzed human pre-miRNA structures and found that the bulges peaked in the 22-position on the 3p-strand. In contrast, the bulges did not peak in a similar position on the 5p-strand (Fig. 5a, b). Interestingly, many pre-miRNA orthologs from numerous organisms retained the 22-bulge in their structure (Fig. 5c). This suggests that it might play an important role in the function of miRNAs. We selected three pre-miRNAs, which are known to contain the 22-bulge (i.e., pre-mir-143, pre-mir-376a-2, and pre-mir-410), and mutated their 22-bulge (Fig. 5d). We found that DICER cleaved 22-bulge-pre-miRNAs, including pre-mir-143 and pre-mir-376a-2, with higher efficiency and accuracy than nobulge-pre-miRNAs (Fig. 5e, f, and Supplementary Fig. 5a). Interestingly, DICER cleaved the 22-bulge-containing pre-mir-410 (WT) at DC21 but shifted the cleavage site to DC22 upon removal of the 22-bulge (Fig. 5e, g). In addition, we demonstrated that the 22-bulge also increased the DC21 cleavage of the DICER-TRBP complex on pre-mir-143 (Supplementary Fig. 5b). These findings again indicate the critical role of the 22-bulge in DICER cleavage activity and thus in miRNA biogenesis.

### Single nucleotide polymorphism-associated 22-bulges affect the cleavage sites of DICER

RNA editing, RNA modification, or DNA mutations (such as single nucleotide polymorphisms; SNPs), might alter the 22-bulge in pre-miRNAs, resulting in changes in miRNA biogenesis. Therefore, we analyzed miRNASNPs^38^, which contained SNPs and mutations in genes coding for miRNAs. We found 194 SNPs and mutations that result in the appearance or disappearance of the 22-bulge in various pre-miRNAs (Supplementary Fig. 5c). We demonstrated that the SNP-introduced 22-bulge in pre-mir-216a significantly increased the cleavage accuracy of DICER. We also showed that an SNP in pre-mir-376a-2, which switched the bulge from the 22-position (22-B) to the 21-position (21-B), reduced the cleavage accuracy of DICER (Fig. 5h–j). In contrast, an SNP in pre-mir-337 shifted the bulge from the 23-position (23-B) to the 22-position (22-B), enhancing the cleavage accuracy of DICER. DICER-TRBP also showed a similar cleavage pattern as DICER on pre-mir-216a and pre-mir-337 (Supplementary Fig. 5d). In addition, the 1-nt and 2-nt bulges had a better effect on the DICER cleavage than the more prominent bulges containing 3 or 4 nt (Supplementary Fig. 5e–g).

We then expressed the WT and SNP pri-miRNAs (i.e., pri-mir-216a and pri-mir-376a-2) in the HCT116 cells transfected with the pri-miRNA-coding DNA-containing pCDNA3, and then we conducted small-RNA sequencing. Consistent with the in vitro cleavage results, we found that the 22-bulge-containing pre-miRNAs increased DC21-miRNAs in the human cells (Fig. 5k). To exclude the possibility that these SNPs might have altered the cleavage sites of the Microprocessor in these pri-miRNAs, we analyzed the 5p-end of the 5p-miRNAs and 3p-end of the 3p-miRNAs and showed that the cleavage sites of Microprocessor were similar between the WT and SNP-pri-miRNAs (Supplementary Fig. 5h). Consistent with shRNAs, DICER also showed the higher cleavage accuracy and efficiency in pre-mir-216a containing 22-bulge on 3p-strand (22-B 3p) than 22-bulge on the 5p-strand (22-B 5p) or 22-mismatch (22-M) (Supplementary Fig. 5i–l). These data indicate the importance of the 22-bulge in governing the accuracy of DICER cleavage and thereby controlling miRNA biogenesis.

### The single cleavage mechanism of DICER

**Fig. 6:**
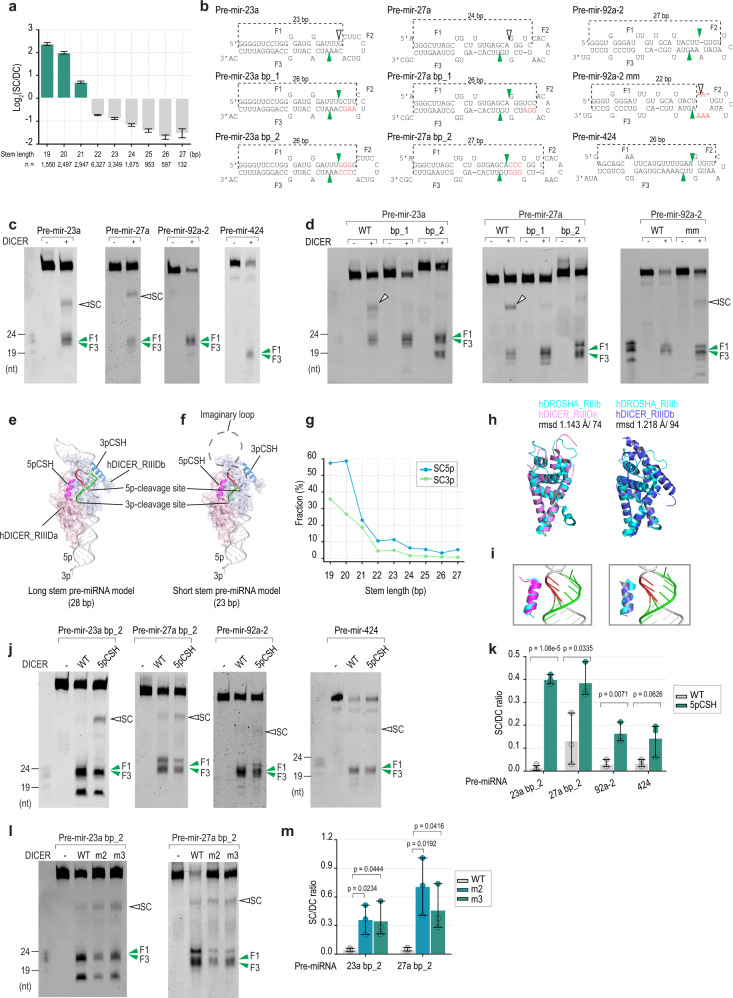
The single cleavage of DICER. **a** The relative single cleavage (SC) of DICER was calculated as the ratio of SC to DC. The number of variants for each stem length: *n* = 1550 (19 nt); *n* = 2497 (20 nt); *n* = 2947 (21 nt); *n* = 6327 (22 nt); *n* = 3349 (23 nt); *n* = 1675 (24 nt); *n* = 953 (25 nt); *n* = 597 (26 nt); *n* = 132 (27 nt). The error bars were presented with 95% confidence intervals. **b** Human pre-miRNAs and their variants. The mutated nt are shown in red. The stem length of pre-miRNAs was the number of base pairs and mismatches from their 5'-end to their apical loop. **c**, **d** The in vitro DICER cleavage assays for human pre-miRNAs and their variants. The assays were repeated three times. **e**, **f** The two RNase III domains of human DICER (PDB: 5ZAM)^10^ were fitted with the RNA duplex. The RIIIDs of DICER from PDB: 5ZAM^10^ were superimposed on the RIIIDs of DROSHA from PDB: 6V5B^39^ or PDB: 6LXD^40^. The RNA duplex was a part of a pri-miRNA in PDB: 6V5B^39^. The RIIIDa of DICER is in light pink, and the 3pCSH is in violet. The dashed line indicates the anticipated loop of the pre-miRNA. **g** The fractions of the shRNA variants sharing the same stem length and exhibiting 3p-strand or 5p-strand SC. **h** Superimposition of the RIIIDb of DROSHA and the RIIIDa or RIIIDb of DICER. DROSHA_RIIIDb is in cyan (PDB: 6V5B)^39^, DICER_RIIIDa is in violet (PDB: 5ZAM)^10^, and DICER_RIIIDb is in blue (PDB: 5ZAM)^10^. The root-mean-square deviation (rmsd) and the number of aligned residues are shown. The superimposition and figures were drawn using PyMOL (http://www.pymol.org). **i** Close-up view of the possible interaction between 3pCSH and RNA. **j**, **l** The in vitro DICER cleavage assays. **k**, **m** Bar graphs showing ratios of the SC to the double-cut product (F1) band density (SC/DC ratios) from three repeated experiments as shown in (**j,** **l**). In (**k**, **m**), a two-tailed t-test calculated the p-values. The error bars were presented with 95% confidence intervals. Source data are provided as a Source Data file.

We calculated the SC/DC ratio of the shRNA variants and discovered that the short-stem shRNAs exhibited a higher SC level than the long-stem shRNAs (Fig. 6a). In addition, shRNAs of the same length but containing different loop sizes had similar SC/DC levels (Supplementary Fig. 6a). This suggested that the stem length but not the loop size is a critical factor for single cleavage activity. We then tested the DICER cleavage of some pre-miRNAs that contained long or short stems. The stem length of pre-miRNAs was the number of base pairs and mismatches from their 5p-end to their apical loop. We found that DICER exhibited a single cleavage on two short-stem pre-miRNAs, pre-mir-23a (stem length = 23 bp) and pre-mir-27a (stem length = 24 bp), but not on two long-stem pre-miRNAs, pre-mir-92a-2 (stem length = 27 bp) and pre-mir-424 (stem length = 26 bp) (Fig. 6b, c and Supplementary Fig. 6b). We conducted DICER in vitro cleavage of different variants that contained long or short stems using pre-mir-23a, pre-mir-27a, and pre-mir-92a-2 backbones. Interestingly, the short-stem variants but not the long-stem variants exhibited single cleavages (Fig. 6b, d, and Supplementary Fig. 6c).

We superimposed the RIIIDs of the human DICER structure (PDB: 5ZAM^10^) onto those of the human DROSHA-RNA structure (PDB: 6V5B^39^ and PDB: 6LXD^40^). The resulting structural model suggested that the 5pCSH (5p-strand cleavage supporting helix) in the DICER RIIIDa might interact with the 5p-strand of pre-miRNAs, and thus support 5p-strand cleavage by its RIIIDb (Fig. 6e, f). It also suggested that the 3pCSH (3p-strand cleavage supporting helix) in the DICER RIIIDb might interact with the 3p-strand of pre-miRNAs and thus help the 3p-strand cleavage by its RIIIDa (Fig. 6e, f). However, in the latter case, the lengths of the shRNAs are typically shorter than 25 bp, so the 3pCSH might be nonfunctional for many substrates. Consistent with this, we found that DICER had more 5p-single cleavages than 3p-single cleavages on shRNAs (Fig. 6g and Supplementary Fig. 6d).

In a previous study with human DROSHA, we revealed that the 3pCSH in RIIIDb of DROSHA supports its 3p-cleavage^41,42^. Here, we compared the structure of the DROSHA RIIIDb with those of the DICER RIIIDa and RIIIDb. Interestingly, we found that the structure and sequence of the 3pCSH of the DROSHA RIIIDb are similar to the 5pCSH of the DICER RIIIDa than the 3pCSH of DICER RIIIDb (Fig. 6h, i, and Supplementary Fig. 6e). In addition, the N-terminal parts of the 5pCSH and 3pCSH of DICER seemed to contact the RNA duplex (Fig. 6h, i). In contrast, the 3pCSH of the DICER RIIIDb was tilted outward from the RNA duplex (Fig. 6h, i). Therefore, the 3pCSH in the RIIIDb of DROSHA and the 5pCSH in the RIIIDa of DICER might serve a similar role and support the cleavage of the other RIIIDs in the same protein.

We subsequently purified a DICER mutant protein containing mutations in the putative RNA-binding residues in the 5pCSH (Supplementary Fig. 6f, g) and tested its cleavage activity. We found that the 5pCSH mutant generated more single cleavage products on the base-paired variants (bp_2) when compared with the WT (Fig. 6j, k). These findings indicate that the interaction between the 5pCSH and RNA was critical for ensuring the double cleavage of DICER. Next, we found that the mutations in the 3pCSH increased the SC/DC ratio of DICER cleavage in the long stem pre-miRNAs more significantly than the short stem pre-miRNAs (Supplementary Fig. 6i–l).

In addition, we examined the single cleavage activity of DICER that had mutations in its dsRBD (Fig. 6l, m). In accordance with human DROSHA, we also found that the dsRBD-mutated DICER increased the level of the single cleavages (Fig. 6l, m), suggesting that it is the dsRBD of DICER, which supports its double cleavage activity.

## Discussion

The effect of the USL in shRNAs and pre-miRNAs on DICER cleavage is a challenge to investigate since the enzyme cleaves these RNAs in the middle of the USL region. After cleavage, each shRNA or pre-miRNA is split into three different fragments. In cleavage assays that are conducted with a mixture of many different one-loop shRNA or pre-miRNA sequences, it is impracticable to reconstruct the three fragments to form the original shRNA molecules. For this reason, we utilized two-loop shRNAs, in which one loop served as a barcode. This design allowed us to align the cleaved fragments with the original substrates. Thus, we could comprehensively show that DICER has different levels of efficiency, accuracy, and single cleavage ability on different structural USLs. This shRNA design helps study other RNase III enzymes that cleave the USL region, such as DCL1 in plants and other DICERs in different organisms.

**Fig. 7:**
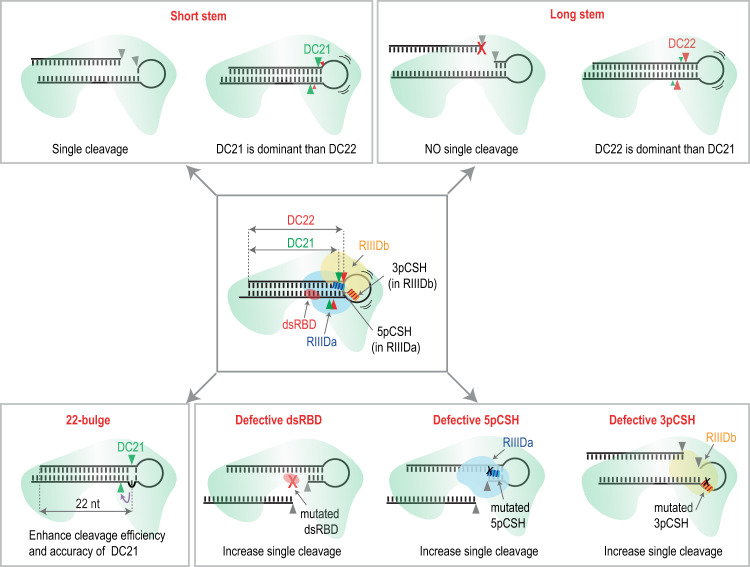
Different cleavage patterns of DICER on pre-miRNAs or shRNAs. DICER mainly cleaves pre-miRNAs or shRNAs at position 21 (DC21) or 22 (DC22) from the 5p-end of the stem. DICER cleaves short-stem pre-miRNAs (19–22 bp) more at DC21 than DC22 and exerts single cleavage at 5p-strand. In contrast, DICER mainly cleaves the long-stem pre-miRNAs (23–27 bp) at DC22. In addition, DICER also exhibits less single cleavage on the long-stem pre-miRNAs. The 22-bulge on the 3p-strand of pre-miRNAs or shRNAs enhances both cleavage efficiency and accuracy of DICER at DC21. Multiple protein-RNA interactions ensure the success of the DICER double cleavage. The defects in the dsRBD domain cause the single cleavage on either 5p- or 3p-strand. The defects in 5pCSH and 3pCSH lead to single cleavage on the 3p- and 5p-strand, respectively.

In shRNA-based gene-silencing technology, the stems of shRNAs make base pairs with their target mRNAs, and thus the stem sequences of shRNAs are variable and determined by the target mRNAs. Currently, shRNAs are designed to contain the stem as an RNA duplex and have a loop of ≥ 6 nt^24^. The addition of an internal or apical loop 2 nt from the cleavage site of DICER enhances the accuracy of DICER action for some shRNAs^20^. Our findings verify the contribution of such a loop in supporting DICER cleavage at DC21 for many shRNAs (Fig. 7). However, our study shows that the vast majority of shRNAs are still not cleaved accurately even when they contain a loop 2 nt from the cleavage site. This study finds that the long stem length of shRNAs increases the DC22 cleavage, which is consistent with the previous observation showing that DICER mainly released 22-nt products from cleaving long double-stranded siRNA precursors^43,44^.

We showed by conducting HT processing assays that an RNA element, the 22-bulge, increased the accuracy and efficiency of DICER cleavage on shRNAs containing different stem sequences (Fig. 7). In addition, the 22-bulge was also shown to enhance the DICER cleavage activity in all of the human pre-miRNAs tested and increased the gene-silencing efficiency of several shRNAs analyzed. Many previous studies suggested a method to design the optimal stem (siRNA) sequences for targeting mRNAs and optimal shRNA-embedded pri-miRNA backbones for better KD effect^24,45,46^. It would be interesting to see if the 22-bulge found in this study and the optimal siRNA sequence can be integrated into the optimal pri-miRNA backbones to increase shRNA's KD efficiency further. Since each siRNA resulting from an shRNA functions to silence mRNAs, the siRNA might also function as a miRNA that targets the endogenous mRNA using the miRNA-mRNA base-pairing mechanism. The resulting few siRNA sequences that arise from inaccurate DICER cleavage on an shRNA might off-target more mRNAs than one siRNA sequence following accurate DICER cleavage. Therefore, the 22-bulge refines DICER cleavage, and as a result, it might reduce the off-target effects caused by shRNAs. Our findings, therefore, provide a foundation for designing more accurate and efficient shRNAs.

Previous studies demonstrate the influence of bulges occurring in various pri-miRNAs on miRNA expression^47–49^. Here we show that the 22-bulge also plays an essential role in controlling the expression of cellular miRNA via enhancing the efficiency and accuracy of DICER cleavage on human pre-miRNAs. The accuracy of DICER cleavage is particularly essential for 3p-miRNAs since inaccurate cleavages might produce 3p-miRNAs containing different seed sequences. Here, we also identified many SNPs that altered the presence of the 22-bulge in many pre-miRNAs or modified the cleavage site and efficacy of DICER. It will be interesting to investigate if any of the SNPs cause cellular defects or human diseases. It is also important to identify mechanisms (such as RNA-editing and RNA-modifications) that alter the 22-bulge, and in this way control miRNA biogenesis.

In this study, we also identified the SC activity of DICER, which can occur on either the 5p or 3p-strand of pre-miRNAs (Fig. 7). Our finding is consistent with a previous report, which demonstrated the SC of DICER on several tested pre-miRNAs^37,50^. Interestingly, we also found that the molecular mechanism of the SC of DICER is somewhat similar to that of DROSHA. We found that the 3pCSH in the RIIIDb of DICER often does not make any contact with pre-miRNAs containing the stem shorter than 25 bp. Therefore, the SC3p of DICER on many pre-miRNAs is not efficient, leading to SC5p. In accordance with this, in our HT analysis, we found that the SC5p of DICER occurs more frequently than SC3p. In humans, these two RNase III enzymes are thought to share a common origin. The SC of DICER has previously been found in Wilms tumor-causing DICER mutations^51–54^. Therefore, it would be of interest to study how this activity controls miRNA biogenesis in some cellular contexts and human diseases such as Wilms tumor. We recently discovered that DROSHAs from different organisms also contain SC activity^42^. It would therefore be of interest to investigate if DICER from other animals and plants also exhibits SC activity. In addition, the uncoupling of DC in RNase III was first found in *Escherichia coli*^55–57^. SC and DC in the same hairpin were the reason for the difference in protein expression observed in hairpin-containing mRNAs. Our findings suggest that DROSHA and DICER might use SC activity in different types of RNA in higher organisms, and identifying the SC substrates of DROSHA and DICER in humans and other organisms will be helpful for future investigations.

## Methods

### The expression and purification of DICER and DICER-TRBP

The pXG-DICER and pXGR-TRBP plasmids were gifts from Dr. Narry Kim (Seoul National University, Korea). To express DICER (or DICER-TRBP), pXG-DICER (or pXG-DICER and pXGR-TRBP) was transfected into 100 of 100 mm dishes of the HEK293E cells, and the transfected cells were collected after 3 days of transfection. The cell pellets were dissolved in T500 buffer containing 20 mM Tris-HCl [pH 7.5], 500 mM NaCl, 4 mM β-mercaptoethanol (Thermo Fisher Scientific), 2 μg/ml RNase A (Thermo Fisher Scientific), together with a protease inhibitor cocktail (Thermo Fisher Scientific). After sonication and high-speed centrifugation, the 45 mL of clear cell lysate were obtained and then mixed with 2 mL of Ni-NTA resin (Bio-Rad). The protein-bound resin was sequentially washed with three buffers containing 20 mM Tris-HCl [pH 7.5], 4 mM β-mercaptoethanol, and 2000 mM NaCl (T2000), 0 mM NaCl (T0), or 150 mM NaCl (T150). The resin-bound proteins were eluted from Ni-NTA resin with T150 (20 mM Tris-HCl [pH 7.5], 4 mM β-mercaptoethanol, and 150 mM NaCl) plus 250 mM imidazole. Next, the eluted proteins were loaded on Q Sepharose Fast Flow resin (GE Healthcare). The Q Sepharose beads were washed with T150, and the proteins were finally eluted from Q Sepharose beads by T500-plus buffer containing 20 mM Tris-HCl [pH 7.5], 500 mM NaCl, 10% glycerol, and 2 mM dithiothreitol (DTT) (Sigma-Aldrich).

### High-throughput shRNA cleavage assays

Nine single-stranded DNA (ssDNA) oligos were obtained from Integrated DNA Technologies (IDT). Each ssDNA contained a region containing 32 random nt, which served as a random barcode for data analysis (Supplementary Data 2). The randomized nts were also introduced in the upper stem-loop (USL) region of shRNAs. Each synthesized ssDNA was annealed to R-set6 (PsiI) through a 23-bp-complementary region. The resulting double-stranded DNAs (dsDNAs) were converted into the complete dsDNAs using Klenow (Thermo Fisher Scientific) at 37 °C for 120 min. Next, the Klenow-synthesized dsDNAs were then amplified using F-T7-noGGG and R-set6 (PsiI) primers in the PCR reactions to obtain dsDNAs containing the T7 promoter. Subsequently, 500 ng of T7-containing dsDNAs were digested with PsiI restriction enzyme (Thermo Fisher Scientific) at 37 °C for 120 min. Finally, 200 ng of the PsiI-digested dsDNAs were added in a 20 µL in vitro transcription (IVT) reaction using the MEGAscript T7 transcription kit (Ambion). The IVT-synthesized RNA substrates (TLR) were gel-purified and quantified using NanoDrop 2000 spectrophotometer (Thermo Fisher Scientific). Subsequently, we collected a total of 9 groups of TLR, naming TLR_1 to TLR_9, containing different randomized regions in the USL. The purified RNAs were finally stored at −80 °C for later use.

In high-throughput DICER cleavage assays, five pmol of each of the nine TLR substrates (from TLR_1 to TLR_9) were incubated with four pmol of the purified DICER proteins in 10 μL of the cleavage assay reaction buffer containing 50 mM Tris-HCl (pH 7.5), 150 mM NaCl, 10% glycerol, 0.2 µg/µL BSA, 1 mM DTT, and 2 mM MgCl_2_. After 120 min incubation at 37 °C, the reactions were stopped by adding 10 μL of the 2X-TBE buffer. Next, the resulting mixtures were incubated with 20 μg of proteinase K (Thermo Fisher Scientific) at 37 °C for 15 min, 50 °C for 15 min, heated at 95 °C for 5 min, and immediately chilled on ice. The chilled reaction samples were then analyzed on 12% urea-PAGE, which were later stained with SYBR™ Green II RNA gel stain (Invitrogen) in 10 min. The bands of the original substrates (OS) and cleaved products (DC and SC) were separately sliced and gel-purified.

The purified RNAs (OS, DC, and SC) were first ligated to the 4N-RA3 adapter (/5rApp/NN NNT GGA ATT CTC GGG TGC CAA GG/3ddC/) using T4 RNA Ligase 2, truncated KQ enzyme (NEB, M0373L). The OS and SC RNAs were cloned in a similar scheme as follows. The 4N-RA3-ligated OS and SC were first precipitated using isopropanol. The precipitated RNAs were resolved in the reverse transcription (RT) mixture containing cirRTP primer (/5Phos/NNN NNN GAT CGT CGG ACT GTA GAA CTC TGA AC/iSp18/CCT TGG CAC CCG AGA ATT CCA) and Superscript IV Reverse Transcriptase (Invitrogen). After 60 min incubation at 50 °C, the RT reaction mixture was treated with 0.1 M NaOH and heated at 98 °C for 10 min to degrade the RNAs. The NaOH-treated RT mixture was loaded onto 12% urea-PAGE gel to separate cDNAs from cirRTP primer. The cDNAs were cut from gel and gel-purified. The purified cDNAs were next circularized using CircLigase ssDNA ligase (Epicentre). The circularized cDNAs were separated from linear cDNAs in 18% urea-PAGE gel and gel-purified. The purified circularized DNAs of OS or SC were finally amplified by PCR using RP1 (5'-AAT GAT ACG GCG ACC ACC GAG ATC TAC ACG TTC AGA GTT CTA CAG TCC GA-3') and RPI1 (5'-CAA GCA GAA GAC GGC ATA CGA GAT CGT GAT GTG ACT GGA GTT CCT TGG CAC CCG AGA ATT CCA-3') or RPI2 (5'-CAA GCA GAA GAC GGC ATA CGA GAT ACA TCG GTG ACT GGA GTT CCT TGG CAC CCG AGA ATT CCA-3'), respectively.

The 4N-RA3-ligated DC was separated from unligated DC and free 4N-RA3 in 12% Urea-PAGE and gel-purified. The purified 4N-RA3-ligated DC was ligated with the 4N-RA5 primer (5'-GUU CAG AGU UCU ACA GUC CGA CGA UCN NNN-3') using the T4 RNA ligase 1. The double ligated DC was reverse-transcribed using Superscript IV Reverse Transcriptase and RTP primer (5'-CAA GCA GAA GAC GGC ATA CGA-3'). Finally, the cDNA was amplified by PCR with RP1 and RPI10 (5'-CAA GCA GAA GAC GGC ATA CGA GAT AAG CTA GTG ACT GGA GTT CCT TGG CAC CCG AGA ATT CCA-3').

As a result, we obtained three DNA libraries for each repeat of the HT cleavage assays. Finally, 7 libraries containing the 3 repeats of OS, 2 repeats of DC, and 2 repeats of SC products were sequenced using Illumina NovaSeq 6000 in 150 bp paired-end mode. Sequencing data were deposited under accession GSE182701 at Gene Expression Omnibus (GEO).

### Analysis of high-throughput shRNA cleavage assays

The sequencing samples mentioned above were processed similarly as follows. The adapters were first removed from both ends of the raw reads using cutadapt^58^ (-a TGGAATTCTCGGGTGCCAAGG -A GATCGTCGGACTGTAGAACTCTGAAC -m 10). Then the pair-end reads were then joined together using fastq-join^59^. The fastq_quality_filter (-q 20 -p 90) was used to collect the high-quality reads. Next, the duplicated reads containing the same 4 nt or 6 nt randomized barcodes in both ends were removed by using fastx_collapser (http://hannonlab.cshl.edu/fastx_toolkit/index.html, version 0.0.13). Then, the OS, DC, and SC samples were further processed separately.

The 4-nt randomized barcodes in the 5p-end and 6-nt randomized barcodes in the 3p-end of the OS reads were removed by cutadapt^58^ (cutadapt -u 4 -u -6). Each read of the OS now contained a full-length shRNA sequence (FL-OS) and a 32-nt (32N) barcodes, which were split into 2 segments (FL-OS and 32N) by using cutadapt^58^ (cutadapt -g GCTTGC…GCAAGC -m 32 -M 32 --discard-untrimmed). The 5'-GCTTGC-3'…5'-GCAAGC-3' sequence was the constant regions of 6 base pairs flanking the 32 N barcode loop. As a result, each read of the OS was now shown as a pair of the FL-OS and 32N. Then, BWA^60^ was used to align FL-OS with the reference sequences containing 23,296 possible variants of 9 shRNA subgroups. The perfectly aligned FL-OS sequences were selected. Therefore, each FL-OS and 32N pair was converted into a variant and 32N pair. The collection of all the variant-32N pairs was considered as an “FL-OS/32N-dictionary”. In the 32N-dictionary, any pairs that contained the same 32N sequence shared by more than 2 variants were discarded, and thus the “unique FL-OS/32N-dictionary” was obtained. The raw counts of an FL-OS were a sum of read counts of the FL-OS in the unique FL-OS/32N-dictionary. Only FL-OS that contained more than 30 raw counts were selected for further analysis.

The cutadapt was applied to remove the randomized barcodes in both ends (cutadapt -u 4 -u -4: 4 nt in both ends for DC reads; cutadapt -u 4 -u -6: 4 nt in 5p-end and 6 nt in 3p-end for SC reads). Each resulting read now contained 2 segments: 32N and the cleaved shRNA product (CP). The reads were also split into these 2 segments, CP and 32N. Next, a 32N sequence of a CP/32N pair in the “CP/32N-dictionary” was aligned with all 32N sequences in the “unique FL-OS/32N-dictionary”, so that the CP in that CP/32N was assigned to an FL-OS sequence that contained the aligned 32N sequences. The cleavage sites of a CP were determined by mapping it to its assigned FL-OS using the local alignment mode in the pairwise2 module from the Biopython library^61^. Given that the cleavage sites of a CP were (x, y), x and y are the 5p and 3p cleavage sites counting from the first nt of shRNA variants, respectively. The length of an shRNA variant without 32N was 72 nt. Each CP was classified based on the DICER cleavage sites as below.

I) Double cleavage with 1-nt overhang at x if 19 ≤ x ≤ 23 and y = 71 – x;
II) Double cleavage with 2-nt overhang (DC) at x if 19 ≤ x ≤ 23 and y = 72 – x;
III) Double cleavage with 3-nt overhang at x if 19 ≤ x ≤ 23 and y = 73 – x;
IV) Single cleavage on 5p (SC5p) at x if 19 ≤ x ≤ 23 and 68 ≤ y ≤ 72;
V) Single cleavage on 3p (SC3p) at 72 – y if 0 ≤ x ≤ 4 and 49 ≤ y ≤ 53;
VI) Unknown, otherwise.

For example, DC21 represents all CP having cleavage site (21, 51), SC5p21 represents all CP having cleavage site (21, y) with y from 68 to 72, and SC3p21 represents all CP having cleavage site (x, 51) with x from 0 to 4.

For each repeat, the “cleaved product” samples of DC and SC were merged by taking the average values of the normalized read counts of CP sharing a similar position on the similarly assigned FL-OS in these two samples. The cleavage efficiency scores for each cleavage site in each variant were calculated using the following formula: Cleavage efficiency score of the cleavage site P = log_2_(N_P_ + 0.1) – log_2_(N_S_ + 0.1). The global cleavage efficiency for each variant was calculated using the following formula: log_2_(∑N_p_ + 0.1) – log_2_(N_S_ + 0.1). P is one of the 15 different cleavage sites (DC, SC5p or SC3p at 19 to 23); ﻿N_P_ is the normalized counts of the CP at the cleavage site P; N_S_ is the normalized counts of the -OS variant generated this CP. 0.1 is a pseudocount. The cleavage accuracy scores of the cleavage site P were calculated using the following formula: N_P_/∑N_p_. The cleavage efficiency and accuracy scores were averaged using 2 scores from the 2 repeats of the “cleaved product” samples.

The secondary structure of each variant was predicted by RNAfold (ViennaRNA Package version 2.4.9) using the default parameters^62^. We collected the minimum free energy structure for each variant for further analysis. From 23,296 variants, we selected 21,465 variants containing 6 base pairs between the upper stem-loop and the 32-nt barcode in their structures for further structural analysis. The dot-bracket structures predicted from RNAfold were converted to our custom format using one of six features for each position: L (loop), M (match), S (symmetric mismatch), A (asymmetric mismatch), B (bulge), and T (3'-overhang). There were 97 typical structures among 21,465 variants. The 58 structures containing more than 10 variants identified from the library were selected. The stem length was defined as the number of nt on the 5p-strand from the first base pair of the stem to the apical loop. The base-pairing probabilities of nt in the bulge were calculated using RNAfold^62^.

### Analysis of human pre-miRNAs

The 556 human pre-miRNA sequences were obtained from MirGeneDB v2.0^63^, and their structures were predicted using RNAfold with default parameters^62^. The pre-miRNA structures with multiple loops were excluded from further analysis. The stem length was defined as the number of base pairs and mismatches on the 5p-strand from the first base pair of the stem to the apical loop. The cleavage sites of DICER were determined by the 3p-end of the 5p miRNAs, which were obtained from MirGeneDB^63^. The position of a bulge on the 5p- or 3p-strand was determined by the number of nt from the first base pair at the 5p-end of the stem to the bulge. We calculated the number of pre-miRNAs containing the bulge in the same positions. We then counted the number of the orthologs, which were deposited in MirGeneDB^63^, for each human 22-bulge containing pre-miRNAs. Next, we calculated the number of these pre-miRNA orthologs containing the 22-bulge.

### Identification of the SNPs/mutations introducing and deleting the 22-bulge

Among the 54,488 SNPs and 10,822 mutations collected from miRNASNPs v3^38^, 11,719 SNPs and mutations that occurred in 556 canonical pre-miRNA sequences on MirGeneDB^63^ were selected. The structures of pre-miRNAs containing SNPs or mutations were predicted using RNAfold^62^. Next, the resulting structures of each WT pre-miRNA and its mutant pre-miRNAs were compared. As a result, we identified 194 SNPs and mutations that either introduced or removed the 22-bulge. The detailed information of the 194 SNPs and mutations above was shown in Supplementary Data 3.

### Pre-miRNA substrate preparation

To generate the DNA template used in the IVT for synthesizing shRNAs, 1-cycle PCR was used to extend the T7 promoter oligo that was fully complementary to the ssDNA oligo containing its reverse complementary sequence and shRNA sequence (Supplementary Data 4). Then, 200 ng of dsDNAs were added in a 20 µL IVT reaction and incubated at 37 °C for 10 h. The IVT-synthesized RNAs were gel-purified by 10% urea-PAGE and quantified using NanoDrop 2000 spectrophotometer. The purified RNAs were stored at -80 °C for further uses.

For human pre-miRNAs, RNA sequences were acquired from MirGeneDB^63^. We synthesized dsDNA sequences containing T7 promoter, a Hammerhead ribozyme sequence of 5'-CUG AUG AGU CCG UGA GGA CGA AAC GGU ACC CGG UAC CGU C-3', and a full sequence of pre-miRNAs (Supplementary Data 4). The resulting dsDNAs were used in IVT to synthesize RNAs. The pre-miRNA sequences separated the Hammerhead ribozyme sequence from the synthesized RNAs by the self-cleaving activity of the ribozyme. The cleaved pre-miRNA containing 5'-OH was converted into 5-monophosphate using the T4 PNK enzyme and ATP. Finally, the 5-monophosphate pre-miRNAs were purified using isopropanol.

### In vitro DICER cleavage assay

Five pmol of each RNA substrate were incubated with different amounts of purified DICER proteins in 10 μL of cleavage assay reaction buffer. The exact amounts of DICER were indicated in the figure legends. After 120 min incubation at 37 °C, the reactions were stopped by adding 10 μl of 2X-TBE buffer plus 20 μg of proteinase K (Thermo Fisher Scientific). These resulting mixtures were incubated at 37 °C for 15 min, 50 °C for 15 min, heated at 95 °C for 5 min, and immediately chilled at 4 °C. The chilled reaction samples were finally analyzed on 12% urea-PAGE, later stained with SYBR™ Green II RNA gel stain (Invitrogen) in 10 min.

### Confirmation of in vitro DICER cleavage

From in vitro DICER cleavage assays, the F2 fragments resulting from DICER cleavage were gel-purified. After the RA3 adapter ligation step, the 4N-RA3-ligated F2 RNAs were separated from the unligated F2 RNAs and free 4N-RA3 by 12% Urea-PAGE and gel-purified. The purified 4N-RA3-ligated F2 fragments were ligated with the 4N-RA5 primer using the T4 RNA ligase 1. The final ligation products were reverse transcribed using Superscript IV Reverse Transcriptase and RTP primer. Finally, the cDNA was amplified by PCR using RP1 and RPIx before sequencing.

### Preparation of small RNA library and sequencing

The shRNA plasmids encoding for 22-bulge (or nobulge) variants of shRNAs were co-transfected into the HEK293T cells in a 6-well plate using Lipofectamine 3000 (Thermo Fisher Scientific). After 48 h, the transfected cells were collected, and their total RNAs were extracted using TRIzol reagent (Invitrogen). 5 μg of each total RNA were size-separated in a 15% urea-PAGE, and the gel slices covering the 19–24 nt region were excised. Small RNA libraries from the gel-eluted small RNAs were cloned using NEBNext® Small RNA Library Prep Set for Illumina® (NEB). The resulting cDNAs were amplified using PCR with distinct index primers to produce DNA libraries.

The pCDNA3 plasmids encoding pri-mir-216a, pri-mir-376a-2 or its SNP variants (set 1), or encoding pri-mir-216a variants with different structural features (22-bulge 3p, 22-single mismatch, 22-bulge 5p, or no-bulge) (set 2) were co-transfected into the HCT116 cells in a 6-well plate using Lipofectamine 3000 (Thermo Fisher Scientific) (Supplementary Data 5). After 36 h, the total RNAs were extracted from the transfected cells using TRIzol reagent (Invitrogen). 5 μg of each total RNA were size-separated in a 15% urea-PAGE, and the gel slices covering the 19–24 nt region were excised. Small RNA libraries from the gel eluted small RNAs were cloned using NEBNext® Small RNA Library Prep Set for Illumina® (NEB). The resulting cDNAs were amplified using PCR with distinct index primers to produce DNA libraries.

The DNA libraries of the small RNAs were run using Illumina Nextseq 500. Sequencing data were deposited under accession GSE182700, GSE183552, and GSE192613 at Gene Expression Omnibus (GEO).

### Analysis of small RNA sequencing

We removed the adapters from the raw reads using cutadapt (-a AGATCGGAAGAGCACACGTCT -A GATCGTCGGACTGTAGAACTCTGAAC)^57^ and joined the pair-end reads using fastq-join^59^. The low-quality reads were discarded using fastq_quality_filter (-q 20 -p 90) (http://hannonlab.cshl.edu/fastx_toolkit/index.html, version 0.0.13). The remaining reads were then mapped to the transfected shRNA or pri-miRNA reference sequences using Bowtie2^64^. The reference sequences contain shRNA sequences followed by the 10-nt poly-T tails for shRNA transfection or pri-miRNA variants for pri-miRNA transfection. The unique mapped reads were collected. In the experiments of shRNA transfection, the cleaved reads of DICER at the cleavage site x (DCx) were defined as the mapped reads starting at L - x and ending at L, in which x ranged from 20 to 23, and L was the length of shRNAs without the poly-T tails. The cleavage accuracy of DICER at DC21 was defined as [the cleaved reads at DC21]/[sum of the cleaved reads from DC20 to DC23]. The cleavage accuracy of DICER at DC21 was averaged for 3 repeats.

### Western blotting

The HepG2 cells were transfected with 22-bulge-shRNA or nobulge-shRNA plasmid that targeted mRNAs of TTR. The shRNA plasmids were constructed using pU6-Sp-pegRNA-HEK3_CTT_ins, a gift from David Liu (Addgene plasmid # 132778), as a backbone. After 2.5 days, the transfected cells were harvested and lysed in the T500 buffer. After sonication, the cell lysates were later analyzed in 12% polyacrylamide-SDS gels. The proteins on SDS-gel were transferred to a blot membrane (PVDF, 1620177, Bio-Rad). After transferring, the blot membrane was blocked with a 3% BSA-containing PBS buffer and then incubated with the TTR antibody (66108- 1-Ig, Proteintech) with a dilution factor of 1:1000. The primary antibody-bound membrane was washed with 0.05% PBST buffer and then incubated with a secondary antibody (conjugated to horseradish peroxidase, HRP, Proteintech) with a dilution factor of 1:3000. We also conducted the western blot for tubulin using tubulin antibody (66031- 1-Ig, Proteintech) with a dilution factor of 1:3000. The signal was developed using ECLSuperSignal™ West Femto Maximum Sensitivity Substrate (Thermo Fisher Scientific) and measured using the ChemiDoc system (Bio-Rad).

### Reporter assays

The reporter assays were conducted using the HEK293T cells. The cells were seeded on a 96-well plate and grown in DMEM containing 10% FBS. The shRNA plasmids and the mixture of two reporter plasmids, Luciferase plasmids (75 ng) and Renilla luciferase (25 ng) were transfected to 3 × 10^4^ cells in a well using Lipofectamine 3000 (Thermo Fisher Scientific) (Supplementary Data 6). The exact amounts of shRNA plasmids were indicated in the figure legends. After 48 h, the transfected cells were harvested and lysed in 20 μL of lysis buffer (E1980, Promega). Next, the Firefly and Renilla luciferase activities were assayed using luciferase substrate (E1980, Promega) and measured using a multi-mode reader (flexstation 3 multi-mode microplate reader). The relative expression of FL luciferase was first normalized against that of RL luciferase. The fold repression (knockdown efficiency) was calculated as the ratio of the RL-normalized FL luciferase expression of FL-shRNA to that of the FL-control.

### Reporting summary

Further information on research design is available in the Nature Research Reporting Summary linked to this article.

## Supplementary information

Supplementary Information

Description of Additional Supplementary Files

Supplementary Data 1

Supplementary Data 2

Supplementary Data 3

Supplementary Data 4

Supplementary Data 5

Supplementary Data 6

Reporting Summary

## Data Availability

Pri-miRNAs sequences were collected from MirGeneDB v2.0^63^, SNPs and mutations were collected from miRNASNPs v3^38^. Protein structures were obtained from Protein Data Bank (PDB: 5ZAM, 6V5B, 6LXD). The RNA sequencing data generated in this study have been deposited in the Gene Expression Omnibus database under accession code GSE182700, GSE182701, GSE183552, and GSE192613. The source data underlying Figs. 1c, e, f, i–k, 2e–g, 3h–j, 4b–e, g–i, 5a–c, e–g, i–k, 6c, d, j–m and Supplementary Figs. 1b, c, e, 2c–e, g, h, l, m, 4b–g, i, j, 5a, b, d, f–h, j–l, 6a–c, g–l are provided as a Source Data file. All other data are available from the corresponding author upon reasonable request. Source data are provided with this paper.

## Source data

Source Data

## References

[1] Ameres SL, Zamore PD (2013). Diversifying microRNA sequence and function. Nat. Rev. Mol. Cell Biol..

[2] Friedman RC, Farh KKH, Burge CB, Bartel DP (2009). Most mammalian mRNAs are conserved targets of microRNAs. Genome Res..

[3] Jonas S, Izaurralde E (2015). Towards a molecular understanding of microRNA-mediated gene silencing. Nat. Rev. Genet..

[4] Bartel DP (2018). Metazoan MicroRNAs. Cell.

[5] Ha M, Kim VN (2014). Regulation of microRNA biogenesis. Nat. Rev. Mol. Cell Biol..

[6] Nguyen HM, Nguyen TD, Nguyen TL, Nguyen TA (2019). Orientation of Human Microprocessor on Primary MicroRNAs. Biochemistry.

[7] Gebert LFR, MacRae IJ (2019). Regulation of microRNA function in animals. Nat. Rev. Mol. Cell Biol..

[8] Iwasaki, S. & Tomari, Y. Argonaute-mediated translational repression (and activation). 10.4161/fly.3.3.9025 (2009). 19556851

[9] Lau PW (2012). The molecular architecture of human Dicer. Nat. Struct. Mol. Biol..

[10] Liu Z (2018). Cryo-EM Structure of Human Dicer and Its Complexes with a Pre-miRNA Substrate. Cell.

[11] MacRae IJ, Li F, Zhou K, Cande WZ, Doudna JA (2006). Structure of Dicer and Mechanistic Implications for RNAi. in. Cold Spring Harb. Symposia Quant. Biol..

[12] MacRae IJ, Zhou K, Doudna JA (2007). Structural determinants of RNA recognition and cleavage by Dicer. Nat. Struct. Mol. Biol..

[13] Zhang H, Kolb FA, Jaskiewicz L, Westhof E, Filipowicz W (2004). Single processing center models for human Dicer and bacterial RNase III. Cell.

[14] Bernstein E, Caudy AA, Hammond SM, Hannon GJ (2001). Role for a bidentate ribonuclease in the initiation step of RNA interference. Nature.

[15] Ma E, Zhou K, Kidwell MA, Doudna JA (2012). Coordinated activities of human dicer domains in regulatory RNA processing. J. Mol. Biol..

[16] Wostenberg C (2012). The Role of Human Dicer-dsRBD in processing small regulatory RNAs. PLoS One.

[17] Doyle M (2013). The double-stranded RNA binding domain of human Dicer functions as a nuclear localization signal. RNA.

[18] Park JE (2011). Dicer recognizes the 5′ end of RNA for efficient and accurate processing. Nature.

[19] Tian Y (2014). A phosphate-binding pocket within the platform-PAZ-connector helix cassette of human dicer. Mol. Cell.

[20] Gu S (2012). The loop position of shRNAs and pre-miRNAs is critical for the accuracy of dicer processing in vivo. Cell.

[21] Tsutsumi A, Kawamata T, Izumi N, Seitz H, Tomari Y (2010). Recognition of the pre-miRNA structure by Drosophila-Dicer-1. Nat. Struct. Mol. Biol..

[22] Liu Z, Wang J, Li G, Wang H-W (2014). Structure of precursor microRNA’s terminal loop regulates human Dicer’s dicing activity by switching DExH/D domain. Protein Cell.

[23] Zhang X, Zeng Y (2010). The terminal loop region controls microRNA processing by Drosha and Dicer. Nucleic Acids Res..

[24] Bofill-De Ros X, Gu S (2016). Guidelines for the optimal design of miRNA-based shRNAs. Methods.

[25] Moore CB, Guthrie EH, Huang MTH, Taxman DJ (2010). Short hairpin RNA (shRNA): Design, delivery, and assessment of gene knockdown. Methods Mol. Biol..

[26] Paddison PJ, Caudy AA, Bernstein E, Hannon GJ, Conklin DS (2002). Short hairpin RNAs (shRNAs) induce sequence-specific silencing in mammalian cells. Genes Dev..

[27] Rao DD (2010). Enhanced target gene knockdown by a bifunctional shRNA: A novel approach of RNA interference. Cancer Gene Ther..

[28] Setten RL, Rossi JJ, Han S. ping (2019). The current state and future directions of RNAi-based therapeutics. Nat. Rev. Drug Discov..

[29] Siolas D (2005). Synthetic shRNAs as potent RNAi triggers. Nat. Biotechnol..

[30] Burnett JC, Rossi JJ, Tiemann K (2011). Current progress of siRNA/shRNA therapeutics in clinical trials. Biotechnol. J..

[31] Liu M (2020). Targeting PD-L1 in non-small cell lung cancer using CAR T cells. Oncogenesis.

[32] Giering JC, Grimm D, Storm TA, Kay MA (2008). Expression of shRNA from a tissue-specific pol II promoter is an effective and safe RNAi therapeutic. Mol. Ther..

[33] Xia XG (2003). An enhanced U6 promoter for synthesis of short hairpin RNA. Nucleic Acids Res..

[34] Zhou H, Xia XG, Xu Z (2005). An RNA polymerase II construct synthesizes short-hairpin RNA with a quantitative indicator and mediates highly efficient RNAi. Nucleic Acids Res..

[35] Schopman NCT, Liu YP, Konstantinova P, ter Brake O, Berkhout B (2010). Optimization of shRNA inhibitors by variation of the terminal loop sequence. Antivir. Res..

[36] Nguyen TA (2015). Functional anatomy of the human microprocessor. Cell.

[37] Starega-Roslan J (2011). Structural basis of microRNA length variety. Nucleic Acids Res..

[38] Liu C-J (2021). miRNASNP-v3: a comprehensive database for SNPs and disease-related variations in miRNAs and miRNA targets. Nucleic Acids Res..

[39] Partin AC (2020). Cryo-EM Structures of Human Drosha and DGCR8 in Complex with Primary MicroRNA. Mol. Cell.

[40] Jin W, Wang J, Liu C-PP, Wang H-WW, Xu R-MM (2020). Structural Basis for pri-miRNA recognition by Drosha. Mol. Cell.

[41] Nguyen TL, Nguyen TD, Bao S, Li S, Nguyen TA (2020). The internal loops in the lower stem of primary microRNA transcripts facilitate single cleavage of human Microprocessor. Nucleic Acids Res..

[42] Nguyen TL, Nguyen TD, Nguyen TA (2021). The conserved single-cleavage mechanism of animal DROSHA enzymes. Commun. Biol..

[43] Zhang H, Kolb FA, Brondani V, Billy E, Filipowicz W (2002). Human Dicer preferentially cleaves dsRNAs at their termini without a requirement for ATP. EMBO J..

[44] Starega-Roslan J, Galka-Marciniak P, Krzyzosiak WJ (2015). Nucleotide sequence of miRNA precursor contributes to cleavage site selection by Dicer. Nucleic Acids Res..

[45] Fellmann C (2013). An optimized microRNA backbone for effective single-copy RNAi. Cell Rep..

[46] Knott SRV (2014). A computational algorithm to predict shRNA potency. Mol. Cell.

[47] Li S, Le TNY, Nguyen TD, Trinh TA, Nguyen TA (2021). Bulges control pri-miRNA processing in a position and strand-dependent manner. RNA Biol..

[48] Roden C (2017). Novel determinants of mammalian primary microRNA processing revealed by systematic evaluation of hairpin-containing transcripts and human genetic variation. Genome Res..

[49] Bofill-De Ros X (2019). Structural differences between Pri-miRNA paralogs promote alternative drosha cleavage and expand target repertoires. Cell Rep..

[50] Flores-jasso CF (2009). First step in pre-miRNAs processing by human Dicer. Acta. Pharmacol. Sin..

[51] Heravi-Moussavi, A. et al. Recurrent Somatic DICER1 Mutations in Nonepithelial Ovarian Cancers. 10.1056/NEJMoa1102903**366**, 234–242 (2012). 10.1056/NEJMoa1102903 22187960

[52] Rakheja D (2014). Somatic mutations in DROSHA and DICER1 impair microRNA biogenesis through distinct mechanisms in Wilms tumours. Nat. Commun..

[53] Vedanayagam J (2019). Cancer-associated mutations in DICER1 RNase IIIa and IIIb domains exert similar effects on miRNA biogenesis. Nat. Commun..

[54] Wu M (2013). Biallelic DICER1 mutations occur in Wilms tumours. J. Pathol..

[55] Calin-Jageman I, Nicholson AW (2003). RNA structure-dependent uncoupling of substrate recognition and cleavage by Escherichia coli ribonuclease III. Nucleic Acids Res..

[56] Nicholson AW, Niebling KR, Mcosker PL, Robertson HD (1988). Accurate in vitro cleavage by RNAse in of phosphorothioate-substituted RNA processing signals in bacteriophage T7 early mRNA. Nucleic Acids Res..

[57] Saito H, Richardson CC (1981). Processing of mRNA by ribonuclease III regulates expression of gene 1.2 of bacteriophage T7. Cell.

[58] Martin M (2011). Cutadapt removes adapter sequences from high-throughput sequencing reads. EMBnet. J..

[59] Aronesty E (2013). Comparison of sequencing utility programs. Open Bioinforma. J..

[60] Li H, Durbin R (2010). Fast and accurate long-read alignment with Burrows-Wheeler transform. Bioinformatics.

[61] Cock PJAA (2009). Biopython: Freely available Python tools for computational molecular biology and bioinformatics. Bioinformatics.

[62] Lorenz R (2011). ViennaRNA Package 2.0. Algorithms Mol. Biol.

[63] Fromm B (2020). MirGeneDB 2.0: The metazoan microRNA complement. Nucleic Acids Res..

[64] Langmead B, Salzberg SL (2012). Fast gapped-read alignment with Bowtie 2. Nat. Methods.

