# Supplementary material for: Secondary structure RNA elements control the cleavage activity of DICER
Source: Nat Commun. 2022 Apr 19;13:2138. doi: 10.1038/s41467-022-29822-3 (PMC9018771; doi:10.1038/s41467-022-29822-3)
Supplement: Supplementary file 1 — Supplementary Information [file 41467_2022_29822_MOESM1_ESM.pdf]

Supplementary Information

Secondary structure RNA elements control the cleavage activity of DICER

Trung Duc Nguyen, Tam Anh Trinh, Sheng Bao, and Tuan Anh Nguyen

Supplementary Figure 1. The high-throughput DICER cleavage assays

Supplementary Figure 2 Cleavage activity of DICER on different upper stem-loop structures

Supplementary Figure 3 The 22-bulge governs the accuracy and efficiency of DICER cleavage at DC21

Supplementary Figure 4 The 22-bulge enhances DICER activity and the knockdown efficiency of shRNAs

Supplementary Figure 5 The 22-bulge controls the cleavage activity of DICER on human pre-miRNAs

Supplementary Figure 6 The single cleavage of DICER

Supplementary Fig. 1

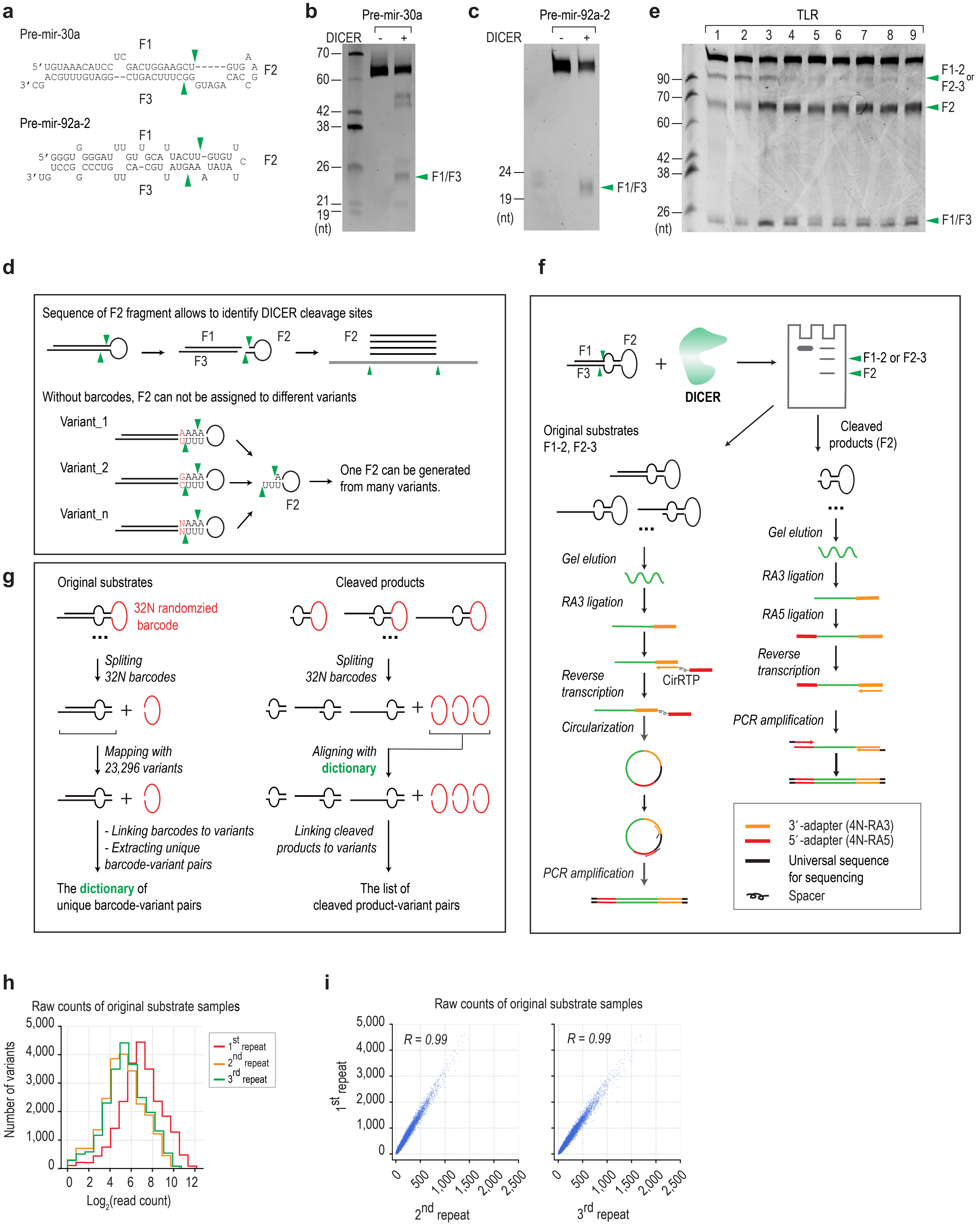

Supplementary Fig. 1. The high-throughput DICER cleavage assays. a The diagrams of pre-mir-30a and pre-mir-92a-2. The green arrowheads indicate the cleavage sites of DICER. **b, c** The pre-miRNA cleavage assays were repeated three times. **d** The high-throughput cleavage assays. The cloning of F2 products helps identify the DICER cleavage sites on both strands. If an F2 did not contain the barcodes, it could not be assigned to which variant it was generated from. The randomized nt are shown in red. **e** The DICER cleavage assays from 9 subgroups of randomized shRNAs were repeated three times. **f** The RNA cloning scheme for the high-throughput shRNA cleavage assays. The original substrates (TLR) and SC products, purified from the urea-PAGE, were ligated to the 3′-adapter. The RNA substrates were converted into cDNAs using an RT primer, cirRTP. The resulting cDNAs were circularized using Circligase. The circularized cDNAs were finally amplified by PCR using the pairs of sequencing primers. In parallel, the gel-purified F2 fragments were ligated with 3′-adapter and 5′-adapter sequentially. The ligated F2 fragments were converted into cDNAs using the R-RA3 primer. The resulting cDNAs were eventually amplified by PCR using the pairs of sequence primers, which had different indexes from those used for the substrates. **g** The pipeline of mapping the cleaved products to their shRNA variants using 32-nt randomized barcodes. The linkage of the 32-nt randomized barcodes and the original substrate or the cleaved product reads in each sample were extracted. Then, we mapped the original substrate with the reference sequences containing 23,296 variants. We generated the dictionary of variants with 32-nt randomized barcodes. In this dictionary, we only collected the unique barcode-variant pair so that a barcode belonged to only one variant. We used the unique barcodes in this dictionary to align the cleaved products to the reference sequences. **h** Plots showing the distribution of barcode numbers and read counts for the original substrates from the high-throughput cleavage assays. The distribution of $\log_2$(read counts) are shown. **i** The reproducibility of the high-throughput cleavage assays. The number of read counts for each variant of the original substrates were obtained from NGS analysis. R is Pearson's correlation coefficient. Source data are provided as a Source Data file.

Supplementary Fig. 2

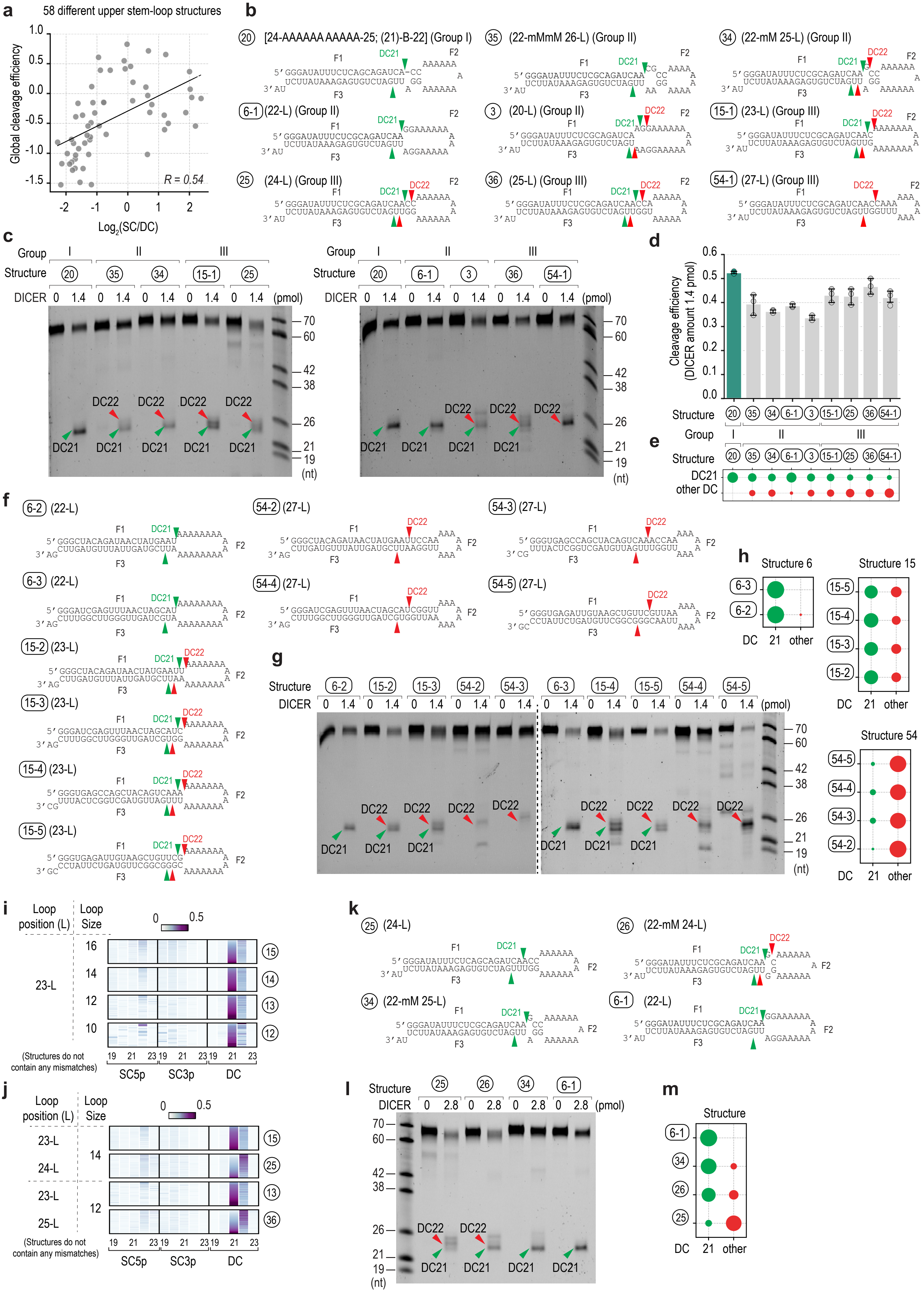

Supplementary Fig. 2 Cleavage activity of DICER on different upper stem-loop structures. **a** The scatter graph of 58 shRNA structures shows the moderate positive correlation between the global cleavage efficiency and the SC/DC ratios. R is Pearson's correlation coefficient. **b** The structures and sequences of the variants containing different structures in I, II, and III groups shown in Fig. 2c. **c** The in vitro DICER cleavage assays for RNAs in (**b**). **d, e** The efficiency and accuracy of DICER cleavage in (**c**) were calculated. The cleavage efficiency of DICER in (**d**) was calculated as a ratio of the cleaved product at DC21 or DC22 to the original substrate, n=3 independent experiments. The error bars were presented with 95% confidence intervals. The cleavage accuracy of DICER in (**e**) was calculated as a ratio of the cleaved product at DC21 to the cleaved products at other positions. **f** The structures and sequences of the variants share the same structures of 6, 15, or 54 but different sequences. **g** The in vitro DICER cleavage assays for RNAs in (**f**). The assays were repeated three times. **h** The cleavage accuracy of DICER in (**g**) was calculated. **i** The cleavage accuracy scores of DICER at DC21 in shRNAs containing the same loop positions but different loop sizes. **j** The cleavage accuracy scores of DICER at DC21 in shRNAs containing the same loop sizes but different loop positions. **k** The structures and sequences of 25, 26, 34, and 6. **l** The in vitro DICER cleavage assays of RNAs in (**k**). The assays were repeated three times. **m** The accuracy of DICER cleavage in (**l**) was calculated. Source data are provided as a Source Data file.

Supplementary Fig. 3

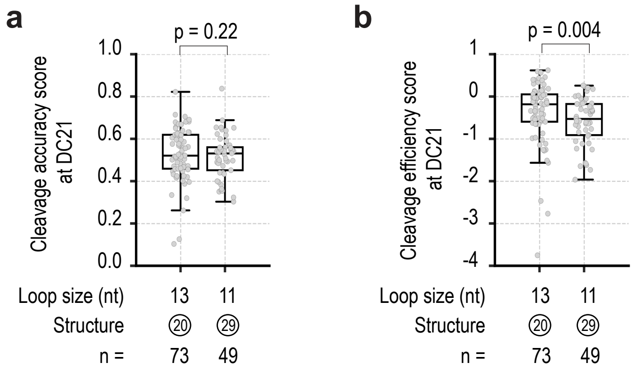

Supplementary Fig. 3 The 22-bulge governs the accuracy and efficiency of DICER cleavage at DC21. a, b The cleavage accuracy and efficiency scores of the 22-bulge variants containing different loop sizes are shown in (**a**) and (**b**), respectively. Structure 20: n = 73 variants, structure 29: n = 49 variants. The center line is median, the lower and upper bounds of the box are the 25th and 75th percentiles, whiskers show 1.5x the interquartile extending from the bounds of the box, minima is the minimum value, and maxima is the maximum value. Individual data values are shown as dots. The p-values were calculated by two-sided Wilcoxon rank-sum tests.

Supplementary Fig. 4

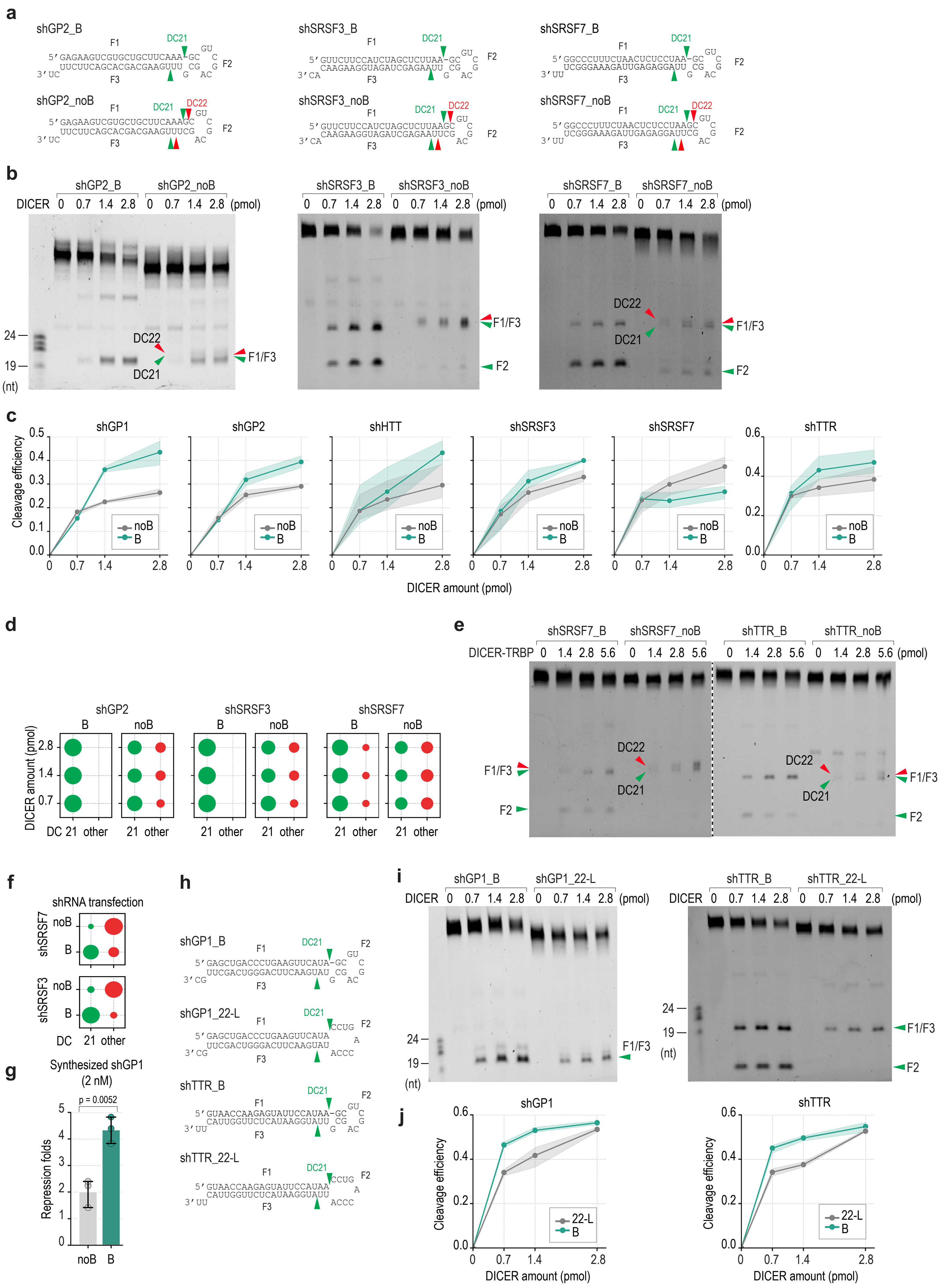

Supplementary Fig. 4 The 22-bulge enhances DICER activity and the knockdown efficiency of shRNAs. a The 22-bulge and nobulge-shRNAs containing different siRNA sequences and sharing the same USLs. **b** The in vitro DICER cleavage assays for 22-bulge and nobulge shRNAs. **c** The cleavage efficiency of DICER on 22-bulge and nobulge shRNAs in Fig. 4b and Supplementary Fig. 4b. The cleavage efficiency of DICER was calculated as a ratio of the cleaved product at DC21 or DC22 to the original substrate. The error bands were drawn with 95% confidence intervals. **d** The cleavage accuracy of DICER on the 22-bulge and nobulge shRNAs in (b). The cleavage accuracy of DICER was calculated as a ratio of the cleaved product at DC21 to the cleaved products at other positions. **e** The in vitro DICER-TRBP cleavage assays for 22-bulge and nobulge shRNAs. **f** Confirmation of the DICER cleavages on the shRNAs in human cells. The siRNAs resulting from cellular shRNAs were sequenced by NGS. **g** The knockdown efficiency of synthetic shGP1. The FL-shRNA (or FL-control) plasmid was co-transfected with Renilla luciferase (RL) plasmid and synthesized shGP1 in the HEK293T cells. The relative expression of FL luciferase was first normalized against that of RL luciferase. The repression folds (knockdown efficiency) were calculated as the ratio of the RL-normalized FL luciferase expression of FL-shRNA to that of FL-control. The dual-luciferase reporter assays were repeated three times. The p-values were calculated by a two-tailed t-test. The error bars were presented with 95% confidence intervals. **h** The structures and sequences of shRNAs. **i** The in vitro DICER cleavage assays of shRNAs in (**h**). **j** The cleavage efficiency of DICER was determined from the shRNA cleavage assays conducted in (**i**). The error bands were drawn with 95% confidence intervals. Source data are provided as a Source Data file.

Supplementary Fig. 5

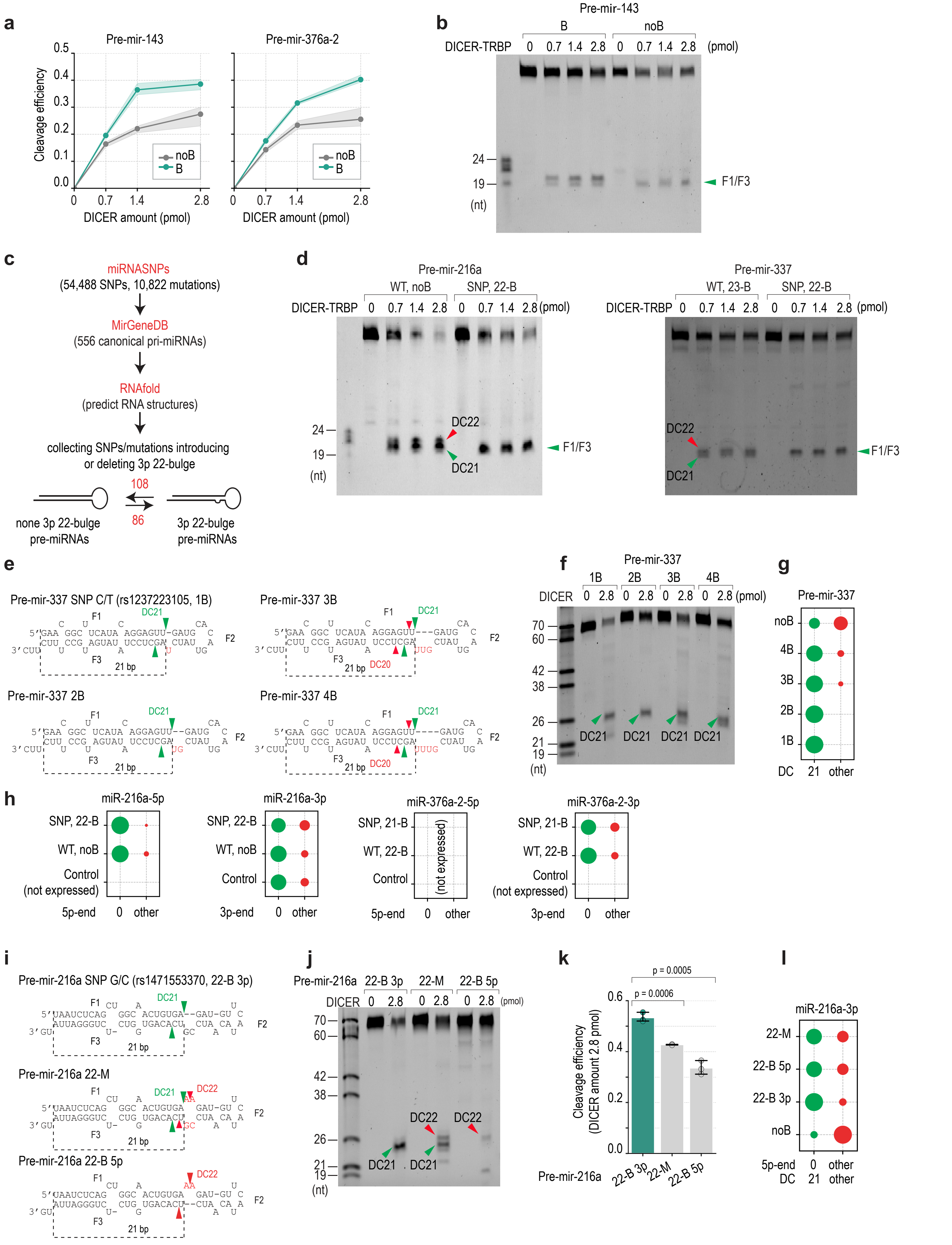

Supplementary Fig. 5 The 22-bulge controls the cleavage activity of DICER on human pre-miRNAs. a The cleavage efficiency of DICER on the 22-bulge pre-miRNAs and nobulge variants in Fig. 5b. The cleavage efficiency of DICER was calculated as a ratio of the cleaved product at DC21 or DC22 to the original substrate. The error bands were drawn with 95% confidence intervals. **b** The in vitro DICER-TRBP cleavage assays for 22-bulge and nobulge pre-miRNAs. **c** The pipeline of identifying SNPs introducing or deleting the 22-bulge. We obtained the SNPs and mutations from miRNASNPs[38] and selected those existing in the 556 canonical pri-miRNAs from MirGeneDB[63]. The structures of WT and SNP/mutated pre-miRNAs were predicted using RNAfold[62]. **d** The in vitro DICER-TRBP cleavage assays for 22-bulge and their SNP pre-miRNAs variants. The assays were repeated three times. **e** The structures and sequences of pre-mir-337 variants containing a different number of nt in the bulge. **f** The in vitro DICER cleavage assays of RNAs in (**e**). The assays were repeated three times. **g** The accuracy of DICER cleavage in (**f**) was calculated as a ratio of the cleaved product at DC21 to the cleaved products at other positions. **h** The 5p-end of 5p miRNAs and 3p-end of 3p-miRNAs were analyzed by NGS. NGS determined the sequences of miRNAs resulting from the ectopic expression of pre-mir-216a and pre-mir-376a-2. The first bases on the miRNAs toward the apical junction were annotated as 0. **i** The structures and sequences of pre-mir-216a variants containing different structural features 22-bulge 3p (22-B 3p), 22-single mismatch (22-mM), or 22-bulge 5p (22-B 5p). **j** The in vitro DICER cleavage assays of RNAs in (**i**). **k** The cleavage efficiency of DICER in (**j**) was calculated as a ratio of the cleaved product at DC21 or DC22 to the original substrate, n=3 independent experiments. The p-values were calculated by a two-tailed t-test. The error bars were presented with 95% confidence intervals. **l** Confirmation of the DICER cleavages on the pre-miRNAs by NGS. NGS determined the sequences of miRNAs resulting from the ectopic expressing pre-mir-216a variants. Source data are provided as a Source Data file.

Supplementary Fig. 6

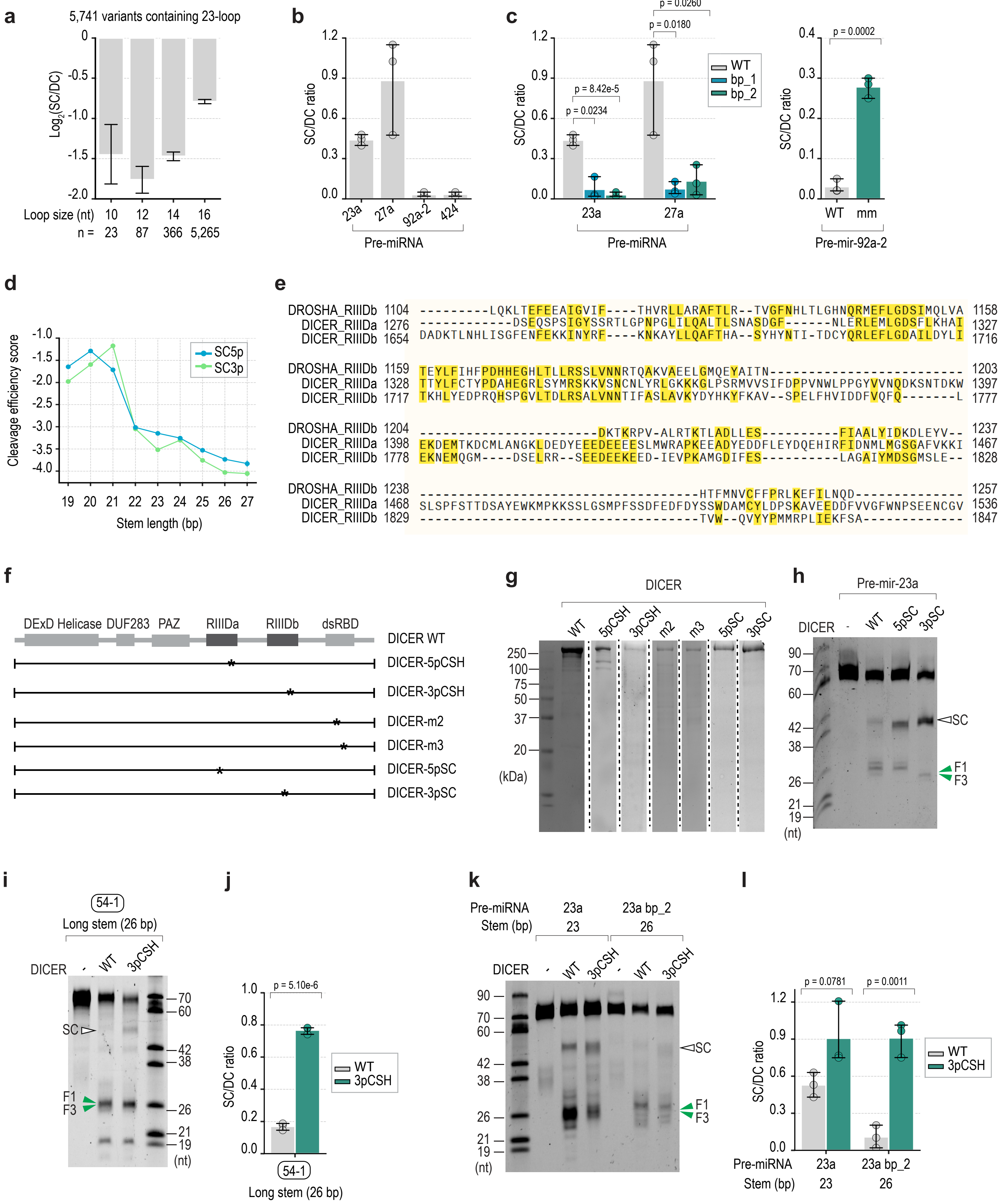

Supplementary Fig. 6 The single cleavage of DICER. a The relative SC levels of DICER on different shRNAs containing 23-loop and different loop-sizes were calculated as the ratio of SC to DC. The number of variants for each loop size: n = 23 (10 nt); n = 87 (12 nt); n = 366 (14 nt); n = 5,265 (16 nt). **b, c** Bar plots show SC/DC ratios of Fig. 6c, 6d. The SC/DC ratio was calculated as a ratio of the single-cut (SC) to double-cut product (F1) band density from the three repeated experiments as shown in Fig. 6c, 6d. The p-values of the two-tailed t-test for the SC/DC ratios measured from three replicates are shown. The error bars were presented with 95% confidence intervals. **d** The SC efficiency scores of DICER on different shRNAs containing different stem-lengths. **e** Alignment of RIIIDb domain of human DROSHA and RIIIDa and RIIIDb of human DICER. **f** Diagram of DICER protein constructs. The numbers indicate the positions of amino acids at the N- and C-terminus of each protein fragment. The asterisks indicate the mutation sites. **g** The quality of the purified WT and mutant DICER proteins was assessed in SDS-PAGE. **h** The in vitro DICER cleavage assays for pre-mir-23a with purified WT and mutant DICER proteins. The assays were repeated three times. **i, k** The in vitro DICER cleavage assays for structure 54 (**i**) or pre-mir-23a variants (**k**) with purified WT and mutant DICER proteins. **j**, **l** Bar graphs showing the SC/DC ratio that was calculated as a ratio of the SC to the double-cut product (F1) band density from three repeated experiments as shown in (**i**) and (**k**). A two-tailed t-test calculated the p-values. The error bars were presented with 95% confidence intervals. Source data are provided as a Source Data file.
