# Supplementary material for: Secondary structure RNA elements control the cleavage activity of DICER
Source: Nat Commun. 2022 Apr 19;13:2138. doi: 10.1038/s41467-022-29822-3 (PMC9018771; doi:10.1038/s41467-022-29822-3)
Supplement: Supplementary file 2 — Description of Additional Supplementary Files [file 41467_2022_29822_MOESM2_ESM.pdf]

Description of Additional Supplementary Files

File Name: Supplementary Data 1
Description: The 58 structures with more than 10 variants in the in vitro high throughput cleavage assays. We predicted the secondary structure of 20,034 shRNA variants using RNAfold. The dot-bracket structure, obtained from RNAfold, was converted into the custom-designed structures in which each nt was assigned with one of these following letters: L (loop), M (match), S (symmetric mismatch), A (asymmetric mismatch), B (bulge), and T (3'-overhang).

File Name: Supplementary Data 2
Description: Primers for making shRNA library and pre-miRNAs

File Name: Supplementary Data 3
Description: SNPs and mutations changing 22-bulge

File Name: Supplementary Data 4
Description: shRNA plasmids

File Name: Supplementary Data 5
Description: Primer sequences

File Name: Supplementary Data 6
Description: Luciferase plasmids
