# Supplementary material for: Secondary structure RNA elements control the cleavage activity of DICER
Source: Nat Commun. 2022 Apr 19;13:2138. doi: 10.1038/s41467-022-29822-3 (PMC9018771; doi:10.1038/s41467-022-29822-3)
Supplement: Supplementary file 9 — Reporting Summary [file 41467_2022_29822_MOESM9_ESM.pdf]

nature research

Corresponding author(s): Tuan Anh NGUYEN

Last updated by author(s): Feb 21, 2022

Reporting Summary

Nature Research wishes to improve the reproducibility of the work that we publish. This form provides structure for consistency and transparency in reporting. For further information on Nature Research policies, see Authors & Referees and the Editorial Policy Checklist.

Statistics

For all statistical analyses, confirm that the following items are present in the figure legend, table legend, main text, or Methods section.

n/a	Confirmed	
☐	☒	The exact sample size (*n*) for each experimental group/condition, given as a discrete number and unit of measurement
☒	☐	A statement on whether measurements were taken from distinct samples or whether the same sample was measured repeatedly
☐	☒	The statistical test(s) used AND whether they are one- or two-sided<br>*Only common tests should be described solely by name; describe more complex techniques in the Methods section.*
☒	☐	A description of all covariates tested
☒	☐	A description of any assumptions or corrections, such as tests of normality and adjustment for multiple comparisons
☐	☒	A full description of the statistical parameters including central tendency (e.g. means) or other basic estimates (e.g. regression coefficient) AND variation (e.g. standard deviation) or associated estimates of uncertainty (e.g. confidence intervals)
☐	☒	For null hypothesis testing, the test statistic (e.g. *F*, *t*, *r*) with confidence intervals, effect sizes, degrees of freedom and *P* value noted<br>*Give P values as exact values whenever suitable.*
☒	☐	For Bayesian analysis, information on the choice of priors and Markov chain Monte Carlo settings
☒	☐	For hierarchical and complex designs, identification of the appropriate level for tests and full reporting of outcomes
☐	☒	Estimates of effect sizes (e.g. Cohen's *d*, Pearson's *r*), indicating how they were calculated

Our web collection on statistics for biologists contains articles on many of the points above.

Software and code

Policy information about availability of computer code

Data collection	Quantification data for gel image collected using Image Lab v6.0.1
Data analysis	Cutadapt v1.15, fastq-join v1.3.1, FASTX-Toolkit v0.0.13, BWA v 0.7.17, Bowtie2 v 2.2.9, RNAfold from ViennaRNA Package v2.4.9, Python 3.7.1, Pairwise2 from Biopython v1.75, Pymol v1.2r3pre, Image Lab v6.0.1.

For manuscripts utilizing custom algorithms or software that are central to the research but not yet described in published literature, software must be made available to editors/reviewers. We strongly encourage code deposition in a community repository (e.g. GitHub). See the Nature Research guidelines for submitting code & software for further information.

Data

Policy information about availability of data

All manuscripts must include a data availability statement. This statement should provide the following information, where applicable:

- Accession codes, unique identifiers, or web links for publicly available datasets
- A list of figures that have associated raw data
- A description of any restrictions on data availability

Pri-miRNAs sequences were collected from MirGeneDB v2.0 [https://mirgenedb.org/], SNPs and mutations were collected from miRNASNPs v3 [http://bioinfo.life.hust.edu.cn/miRNASNP/#!/]. Protein structures were obtained from Protein Data Bank (PDB: 5ZAM [http://doi.org/10.2210/pdb5zam/pdb], 6V5B [http://doi.org/10.2210/pdb6v5b/pdb], 6LXD [http://doi.org/10.2210/pdb6lxd/pdb]). The RNA sequencing data generated in this study have been deposited in the Gene Expression Omnibus database under accession code GSE182700 [https://www.ncbi.nlm.nih.gov/geo/query/acc.cgi?acc=GSE182700], GSE182701 [https://www.ncbi.nlm.nih.gov/geo/query/acc.cgi?acc=GSE182701], GSE183552 [https://www.ncbi.nlm.nih.gov/geo/query/acc.cgi?acc=GSE183552], and GSE192613 [https://www.ncbi.nlm.nih.gov/geo/query/acc.cgi?acc=GSE192613]. The source data underlying Figs. 1c, e, f, i–k, 2e–g, 3h–j, 4b–e, g–i, 5a–c, e–g, i–k, 6c, d, j–m and Supplementary Figs. 1b, c, e, 2c–e, g, h, l, m, 4b–g, i, j, 5a, b, d, f–h, j–l, 6a–c, g–l are provided as a Source Data file. All other data are available from the corresponding author upon reasonable request.

Field-specific reporting

Please select the one below that is the best fit for your research. If you are not sure, read the appropriate sections before making your selection.

☒ Life sciences ☐ Behavioural & social sciences ☐ Ecological, evolutionary & environmental sciences

For a reference copy of the document with all sections, see nature.com/documents/nr-reporting-summary-flat.pdf

Life sciences study design

All studies must disclose on these points even when the disclosure is negative.

Sample size	No sample size calculation was performed. 3 replicates for RNA sequencing of high-throughput shRNA library and other quantitative experiments were performed, which is enough to obtain statistical significance. The sample sizes were indicated in the method section and figure legends.
Data exclusions	No data was excluded.
Replication	3 replicates for RNA sequencing of high-throughput shRNA library and other quantitative experiments.<br>All attempts at replication were successful.
Randomization	No randomization was required and performed in this study.
Blinding	No blinding was performed in this study. All researchers were not blinded during experiments and analysis.

Reporting for specific materials, systems and methods

We require information from authors about some types of materials, experimental systems and methods used in many studies. Here, indicate whether each material, system or method listed is relevant to your study. If you are not sure if a list item applies to your research, read the appropriate section before selecting a response.

Materials & experimental systems

n/a	Involved in the study	
☐	☒	Antibodies
☐	☒	Eukaryotic cell lines
☒	☐	Palaeontology
☒	☐	Animals and other organisms
☒	☐	Human research participants
☒	☐	Clinical data

Methods

n/a	Involved in the study	
☒	☐	ChIP-seq
☒	☐	Flow cytometry
☒	☐	MRI-based neuroimaging

Antibodies

Antibodies used	1:1000 Anti-TTR (Proteintech, 66108- 1-Ig), 1:2000 Anti-tubulin (Proteintech, 66031- 1-Ig). Secondary antibody 1:4000 HRP-conjugated Affinipure Goat Anti-Rabbit IgG (Proteintech, SA00001-2), 1:40000 Anti-mouse IgG (Cell signaling, 7076S)
Validation	The following antibodies were validated by the supplier.<br>Western Blot antibodies:<br>TTR (Proteintech, 66108- 1-Ig)<br>Tubulin (Proteintech, 66031- 1-Ig)

Eukaryotic cell lines

Policy information about cell lines

Cell line source(s)	The cell lines (HCT116, HEK293T, HEK293E) were the gifts from Dr.Narry KIM's lab (Seoul National University, Korea). The HepG2 cell line was the gift from Dr. Angela Ruohao Wu's lab (Hong Kong University of Science and Technology, Hong Kong, China).<br>We could not trace back the commercial source for these cell lines but these cells are available from ATCC.
Authentication	None of the cell lines were authenticated by our facility.
Mycoplasma contamination	All the cell lines were tested negative for mycoplasma contamination.

Commonly misidentified lines
(See ICLAC register)

none used
